# Remodeling of nuclear landscapes during human myelopoietic cell differentiation maintains co-aligned active and inactive nuclear compartments

**DOI:** 10.1186/s13072-015-0038-0

**Published:** 2015-11-17

**Authors:** Barbara Hübner, Mariana Lomiento, Fabiana Mammoli, Doris Illner, Yolanda Markaki, Sergio Ferrari, Marion Cremer, Thomas Cremer

**Affiliations:** Department Biology II, Biocenter, Ludwig Maximilians University (LMU), Grosshadernerstr. 2, 82152 Martinsried, Germany; Department of Life Sciences, University of Modena (Unimore), Modena, Italy; School of Biological Sciences (SBS), Nanyang Technological University (NTU), Singapore, Singapore; Bundeswehr Institute of Radiobiology, Munich, Germany

**Keywords:** Myelopoiesis, Somatic cell differentiation, Nuclear architecture, Active nuclear compartment, Interchromatin compartment, Perichromatin region, Super-resolution microscopy, Electron microscopy, Chromatin domain, Chromatin density classification

## Abstract

**Background:**

Previous studies of higher order chromatin organization in nuclei of mammalian species revealed both structural consistency and species-specific differences between cell lines and during early embryonic development. Here, we extended our studies to nuclear landscapes in the human myelopoietic lineage representing a somatic cell differentiation system. Our longterm goal is a search for structural features of nuclei, which are restricted to certain cell types/species, as compared to features, which are evolutionary highly conserved, arguing for their basic functional roles in nuclear organization.

**Results:**

Common human hematopoietic progenitors, myeloid precursor cells, differentiated monocytes and granulocytes analyzed by super-resolution fluorescence microscopy and electron microscopy revealed profound differences with respect to global chromatin arrangements, the nuclear space occupied by the interchromatin compartment and the distribution of nuclear pores. In contrast, we noted a consistent organization in all cell types with regard to two co-aligned networks, an active (ANC) and an inactive (INC) nuclear compartment delineated by functionally relevant hallmarks. The ANC is enriched in active RNA polymerase II, splicing speckles and histone signatures for transcriptionally competent chromatin (H3K4me3), whereas the INC carries marks for repressed chromatin (H3K9me3).

**Conclusions:**

Our findings substantiate the conservation of the recently published ANC-INC network model of mammalian nuclear organization during human myelopoiesis irrespective of profound changes of the global nuclear architecture observed during this differentiation process. According to this model, two spatially co-aligned and functionally interacting active and inactive nuclear compartments (ANC and INC) pervade the nuclear space.

**Electronic supplementary material:**

The online version of this article (doi:10.1186/s13072-015-0038-0) contains supplementary material, which is available to authorized users.

## Background

Accumulated evidence from different studies argues for an inseparable intertwining between nuclear structure and function [1]. We have recently proposed a model for a functionally defined nuclear organization based on two co-aligned three-dimensional networks: an active and an inactive nuclear compartment (ANC and INC). This model has been experimentally substantiated by super-resolution fluorescence microscopy in mammalian somatic cell lines [2–5] and in bovine preimplantation embryos [6] and can be summarized as follows: Chromosome territories (CTs) are built up from chromatin domain clusters (CDCs), which can perform locally constrained movements and interact dynamically with each other in ways, whose functional implications are still not well understood. The compacted core of CDCs is enriched in repressive histone marks and lined by a peripheral layer of decondensed chromatin, called the perichromatin region (PR). The PR is enriched in epigenetic marks for transcriptionally competent chromatin and represents the chromatin compartment, where transcription, splicing, chromatin replication and DNA repair occur. The PR is co-aligned with a contiguous channel system, the interchromatin compartment (IC), which starts at nuclear pores, permeates the nuclear space between CDCs and serves a role in nuclear import and export functions. More extended sites of this channel system, called IC lacunae, harbor splicing speckles and other nuclear bodies. The PR and the IC together form the ANC, whereas the INC is constituted by the compact interior of CDCs. Constrained motions of CDCs and chromatin loops expanding into the interior of the IC can lead to transient or consistent contacts between random or non-random sites of different CDCs (for a detailed review see [7]).

In this study, we aimed to test the ANC-INC network model during various stages of human myeloid cell differentiation as a naturally occurring somatic cell differentiation system. Myelopoiesis starts from self-renewable pluripotent stem cells, the common progenitor cells. By asymmetric cell divisions resulting daughter cells form lineage restricted precursors with increasing commitment, finally leading to terminally differentiated postmitotic cell types [8, 9]. This maturation process is associated with major changes of the nuclear landscapes [10, 11] and tightly controlled changes of gene expression patterns [12–17]. Proteins located on the cell surface can be used for a refined identification of cells within distinct maturational stages [9, 18].

**Fig. 1:**
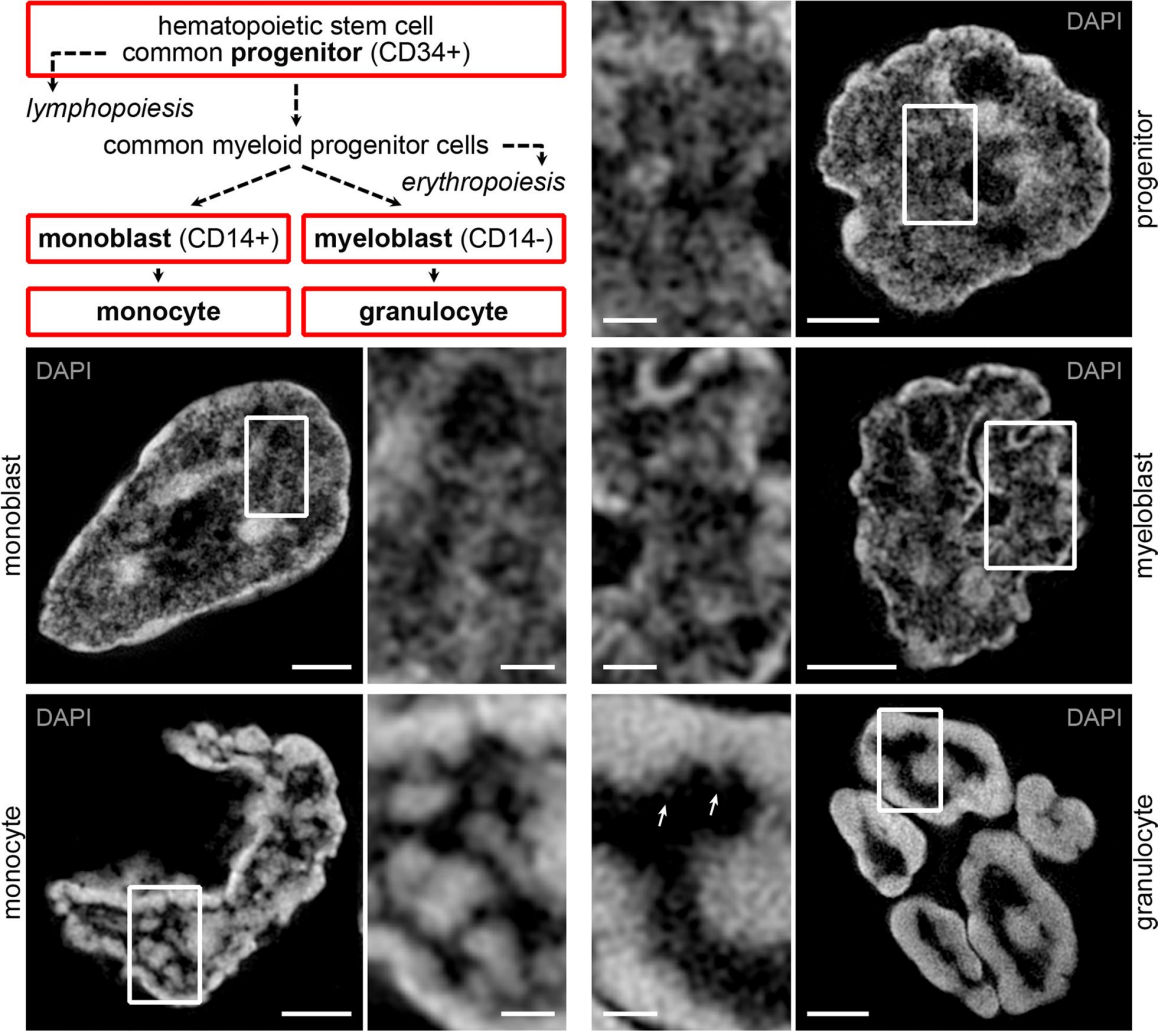
3D-SIM recorded chromatin landscapes of nuclei representing various myelopoietic differentiation stages. *Upper left panel* allocation of the analyzed cell types (framed) within the myeloid differentiation pathway. *Remaining panels* representative xy mid-sections of DAPI stained nuclei recorded with 3D-SIM, exemplifying the transforming global nuclear landscapes during myeloid cell differentiation. A network of chromatin domain clusters (CDCs) permeated by finely branched IC channels is seen in progenitor and precursor cells. Monocytes are characterized by compacted chromatin islets formed by tight aggregations of CDCs embedded within wide IC channels. Granulocytes show a rather uniformly arranged compacted chromatin layer at the nuclear periphery around a central IC lacuna. *Arrows* in *inset* magnification point to few decondensed chromatin sites expanding from the compact chromatin layer. *Scale bars* 2 µm, *insets* 0.5 µm

Myelopoiesis is the part of hematopoiesis involved in the differentiation of multipotent myeloid progenitors into erythrocytes, megakaryocytes, monocytes and granulocytes. For our study, we chose five human cell types from the myeloid differentiation pathway, namely (1) CD34+ cells from umbilical cord blood representing common myeloid and lymphoid progenitors, (2) myeloblasts and monoblasts representing lineage committed precursor cells, as well as (3) monocytes and granulocytes representing differentiated cells (see Fig. 1, upper left panel for an allocation of these cell types within the myeloid differentiation pathway).

Nuclei were imaged by transmission electron microscopy (TEM) and 3D structured illumination microscopy (3D-SIM), a super-resolution fluorescence microscopic approach [19, 20]. 3D-SIM allows optical sectioning with a twofold resolution improvement over conventional fluorescence microscopy in each spatial dimension resulting in an approximately eightfold increased volumetric resolution (for review see [21]). TEM provides a resolution, which is superior to any current approach of super-resolution fluorescence microscopy [22]. However, the capability of 3D-SIM for the simultaneous, high-resolution targeting of differently fluorescence-labeled macromolecules involved in functionally relevant structures, such as RNA polymerase II, nuclear bodies, or epigenetic histone marks, makes this approach an ideal tool for quantitative, high-resolution studies of the nuclear topography of such targets and their spatial nuclear relationships [2, 3, 5, 6].

## Results

### Remodeling of global nuclear landscapes during human myeloid cell differentiation studied with 3D-SIM and TEM

Figure 1 exemplifies typical nuclear phenotypes of DAPI stained progenitor cells (upper panel), monoblast and myeloblast precursor cells (mid panel), and monocytes and granulocytes (bottom panel), represented by xy mid-sections of nuclei acquired with 3D-SIM. Inset magnifications of representative nuclear areas in progenitor and precursor cells reveal a network of chromatin domain clusters (CDCs). CDCs are dispersed throughout the nucleus and pervaded by finely branched IC channels with occasional enlargements into wider IC lacunae. Changes of global nuclear landscapes are most apparent during the transition from precursors toward mature monocytes and granulocytes. Horseshoe-shaped nuclei of monocytes are characterized by aggregations of CDCs into compacted chromatin islets, surrounded by wide interchromatin channels and lacunae. Chromatin in multilobulated nuclei of granulocytes appears mostly restricted to a rather uniformly arranged, densely compacted layer at the nuclear periphery. The interior of each nuclear lobe is filled by an ample contiguous IC lacuna with a few decondensed chromatin loops expanding from the compact chromatin layer toward the interior.

**Fig. 2:**
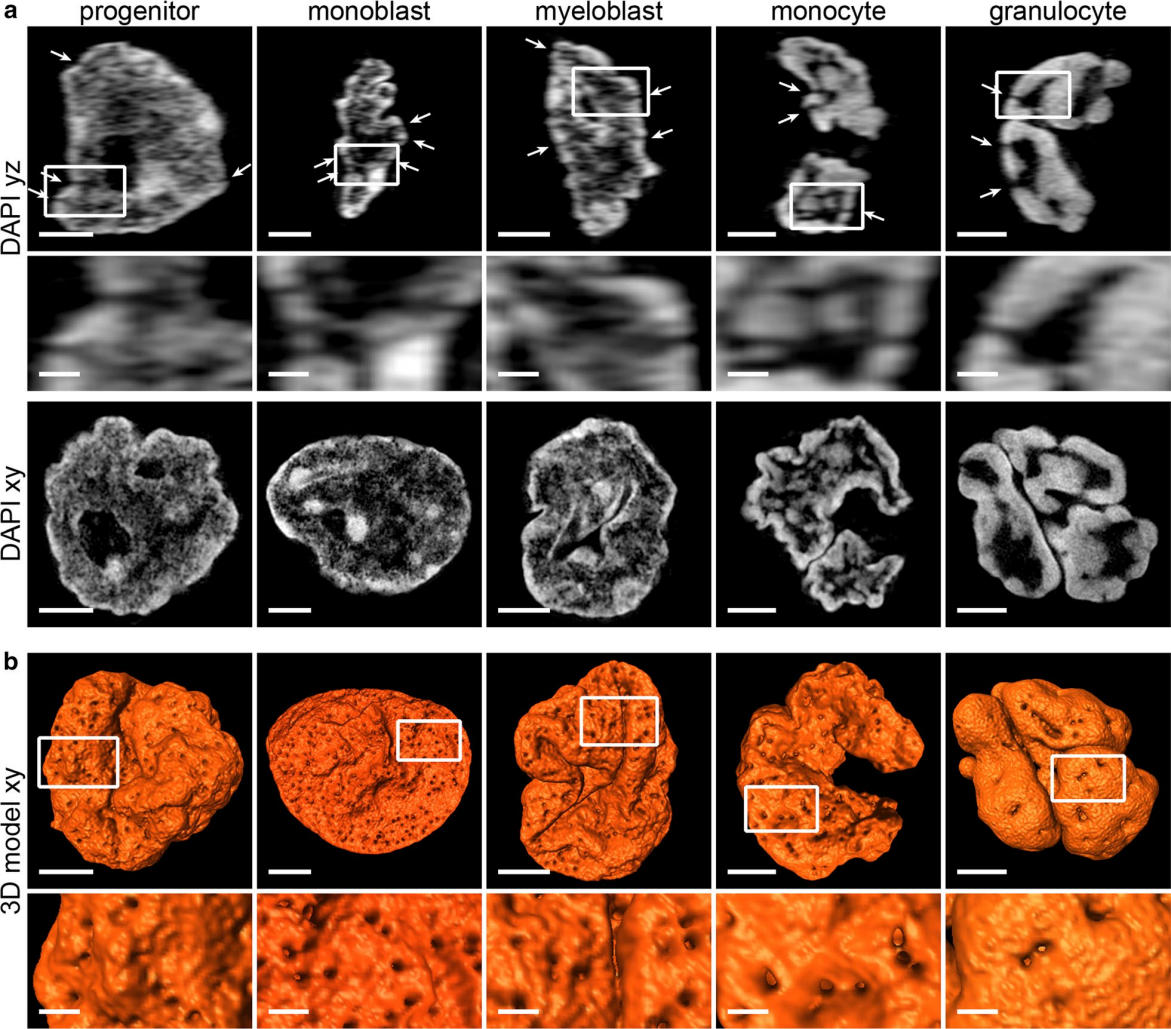
IC channels and nuclear pores. **a** *upper and mid panel:* In all studied cell types IC channels penetrate peripheral (hetero)chromatin toward the nuclear envelope (*arrows*) as shown in vertical (*yz*) sections of 3D-SIM acquisitions. *Bottom panel xy* mid-sections from the respective nuclei for comparison. **b** surface rendering of 3D reconstructions (Amira) of the same nuclei shown in (**a**) reveal small “chromatin holes” mirroring exit point of IC channels (nuclear pores, [23, 24]) at the nuclear envelope. *Scale bars* 2 µm, *insets* 0.5 µm

At all differentiation stages, IC channels penetrate the heterochromatin layer beneath the nuclear envelope (Fig. 2a, arrows). Their exit points appear as little holes on the nuclear surface (Fig. 2b) that were previously shown to be directly connected to nuclear pores [5, 7, 23, 24]. We used these holes, mirroring nuclear pores, to study their topography in 3D reconstructions of 3D-SIM image stacks. Their number is distinctly reduced in monocytes and even more in granulocytes compared to progenitor and precursor cells (Fig. 2b, for quantification see Additional file 1). These images as well as the section galleries shown in Additional file 2 also demonstrate the variations in the global nuclear morphology in the different cell types: nuclei of progenitors exhibit an overall roundish shape with invaginations at the surface. Monoblast and myeloblast nuclei are ellipsoid with typically deep and complex invaginations which can pervade the whole nucleus in myeloblasts. Monocytes are characterized by horseshoe-shaped nuclei with an irregular surface; nuclei of granulocytes are divided into several interconnected lobes.

**Fig. 3:**
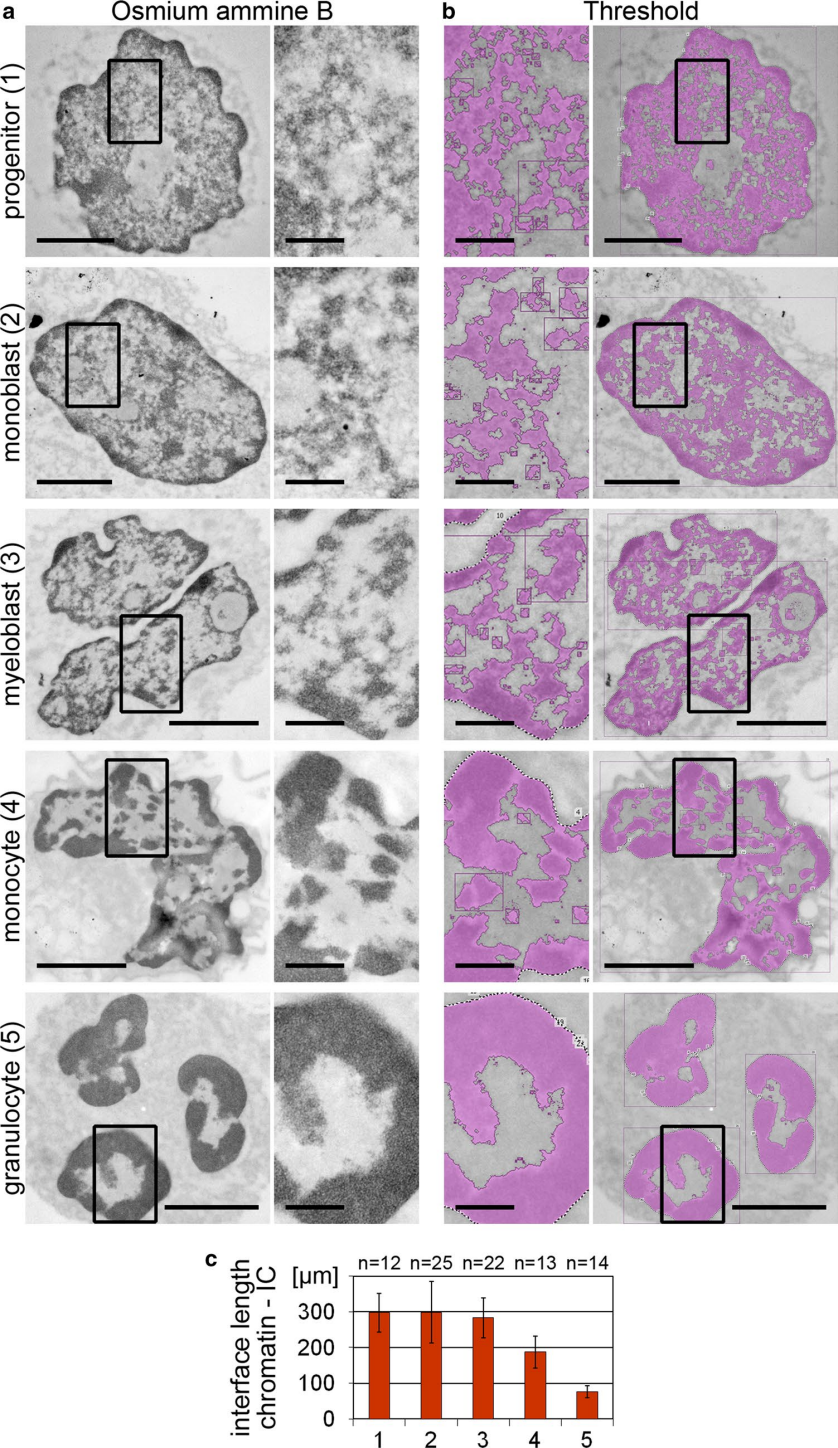
Chromatin landscapes of nuclei of various myelopoietic differentiation stages visualized by TEM in osmium ammine B stained physical sections and assessment of the chromatin/IC interface length. **a** A transition from a fine network of CDCs/IC channels toward a dense and more lump-like pattern was observed with progressive myeloid differentiation. **b** Thresholded masks for the delineation of osmium ammine B stained chromatin (*pink*) and the IC (*gray*) in the respective sections. *Scale bars* 2 µm, *insets* 0.5 µm. **c** The interface length between the thresholded chromatin and the IC is reduced in differentiated cell types (*1* progenitor; *2* monoblast; *3* myeloblast; *4* monocyte; *5* granulocyte). *n* Number of analyzed nuclei; *error bars* standard deviation; *p* < 0.001 for monocytes and granulocytes versus respective precursors and progenitors

Nuclear landscapes recorded with 3D-SIM were compared with landscapes of the same cell types in TEM sections after osmium ammine B staining, a DNA-specific staining procedure (Fig. 3). In line with SIM, TEM images reveal a change of landscapes with progressive differentiation from a fine granular network of CDCs/IC channels in progenitor cells to a dense and more lump-like chromatin pattern in granulocytes (Fig. 3a). However, compared with the range of DAPI staining intensities observed in 3D-SIM images, TEM sections present a more contrasted, black and white appearance of osmium ammine B positive and negative regions. Thresholded TEM images (Fig. 3b) of mid-sections of representative nuclei used to quantitatively determine the extension of the interface between chromatin and the IC as a measure for the IC surface show a significant reduction (p < 0.001) in monocytes and granulocytes compared to their precursors and progenitors (Fig. 3c).

### Nuclear landscapes of human myelopoietic cells delineated by chromatin density classification

**Fig. 4:**
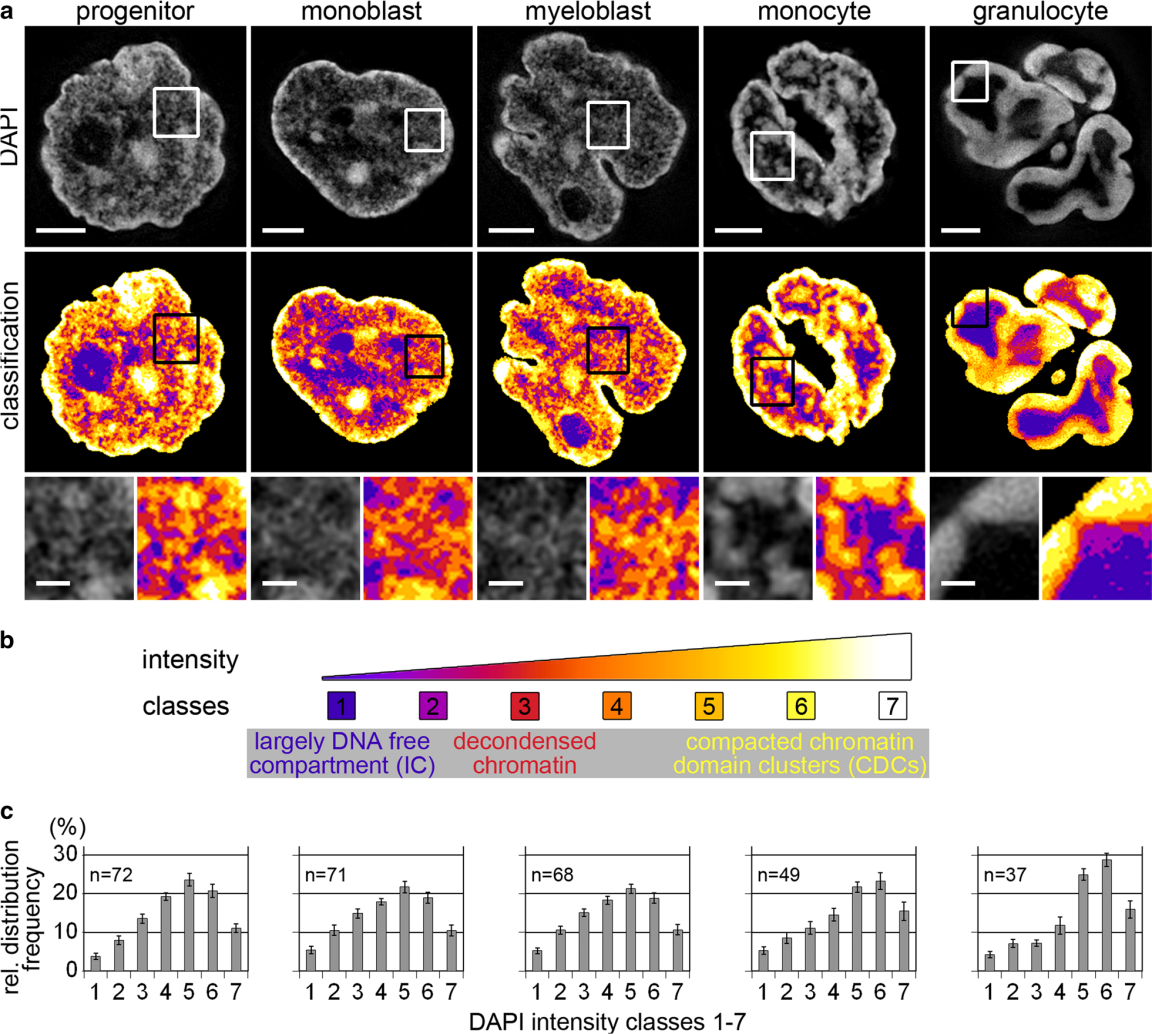
Topological chromatin density mapping of differentiating myelopoietic cell nuclei. **a** *Upper panel:* DAPI stained mid-sections of representative nuclei acquired by 3D-SIM. *Mid panel* same sections after chromatin density classification, based on seven DAPI intensity classes, are displayed in false colors, ranging from class 1 (*blue*) representing pixels close to background intensity, largely reflecting the interchromatin compartment (IC), up to class 7 (*white*) representing pixels with highest density. *Bottom panel* inset magnifications in progenitors, monoblasts and myeloblasts (precursors) reveal a loosely arranged network of small chromatin domain clusters (CDCs) comprising a compacted core part (classes 5–6/7) and a surrounding low-density layer (class 2–4), the perichromatin region. Largely DNA-free class 1 regions meander between CDCs as part of the IC system. Monocytes are characterized by closed up CDCs forming larger islets, surrounded by a decondensed perichromatin layer (classes 2–3). In the lobed granulocyte nucleus, an extended class 1 region, representing the large IC lacuna in the interior of each lobe is lined by a small rim of decondensed chromatin (classes 2–4) at the interface with the highly compacted peripheral chromatin layer (classes 5–7). **b** classification scheme. **c** relative 3D signal distributions of the DAPI intensity classes for each cell type. Note the shift toward higher intensity classes with progressing differentiation but similar values for class 1 despite profoundly different nuclear landscapes. *n* Number of analyzed nuclei; *error bars* standard deviation

A previously described segmentation algorithm [5, 6] was used to obtain 3D chromatin density maps for 3D-SIM acquisitions of whole nuclei from each cell type based on seven DAPI intensity classes of equal intensity variance. DAPI was previously shown to fulfill the requirements as an appropriate marker for global chromatin representation despite its reported binding preference to AT-rich DNA [5]. Figure 4a presents examples of nuclear landscapes before (DAPI, upper panel) and after classification (lower panel) with seven intensity classes plotted in false colors. Class 1 represents regions close to background intensities, assigned to the largely DNA-free interchromatin compartment (IC). Classes 2–4 represent decondensed chromatin of low staining intensity, classes 5–7 the high intensity classes (Fig. 4b). While this classification is a deliberate reduction of the whole range of DAPI intensities (compared, e.g., to 255 gray levels in 8-bit images), it provides a robust tool of statistical comparisons between different samples [5, 6]. In progenitors and precursor cells, the compact core of chromatin domain clusters (CDCs) is represented by aggregations of pixels with highest density assigned to classes 5–7. These compact cores are lined by a layer of pixels assigned to class 2–4, representing the decondensed perichromatin region. Largely DNA-free class 1 regions expand between CDCs as part of the IC system. In the lobed granulocyte nuclei, an extended class 1 region extending into class 2 represents a large IC lacuna in the interior of each lobe, lined by a small rim of decondensed chromatin, while a broad layer of highly compacted chromatin (classes 5–7) resides at the periphery of lobes. Monocytes represent an intermediate state. Quantitative comparisons (Fig. 4c and Additional file 3) demonstrate a significant shift toward higher DAPI intensity classes in nuclei of differentiated cells, while the fraction of class 1 (IC) is similar in all cell types irrespective of their highly divergent global appearance of the IC compartment.

### Linking chromatin density maps with functionally relevant markers

The functional link between nuclear landscapes defined by chromatin density and biologically relevant markers was established by quantitatively mapping the relative spatial distribution of histone H3K4me3, representing transcriptional competent chromatin, and of histone H3K9me3, conveying a silent chromatin state [25], to the seven DAPI intensity classes. In addition we mapped immuno-stained SC35, an integral protein of splicing speckles, involved in co-transcriptional splicing [26] and in transcriptional elongation [27]. We also studied the topography of RNA Polymerase II, phosphorylated in the carboxy-terminal domain either at Ser2 (RNA Pol II Ser2P) or at Ser5 (RNA Pol II Ser5P). RNA Pol II Ser2P is involved in transcriptional elongation, while RNA Pol IISer5P is considered as the transcription initiating form [28].

**Fig. 5:**
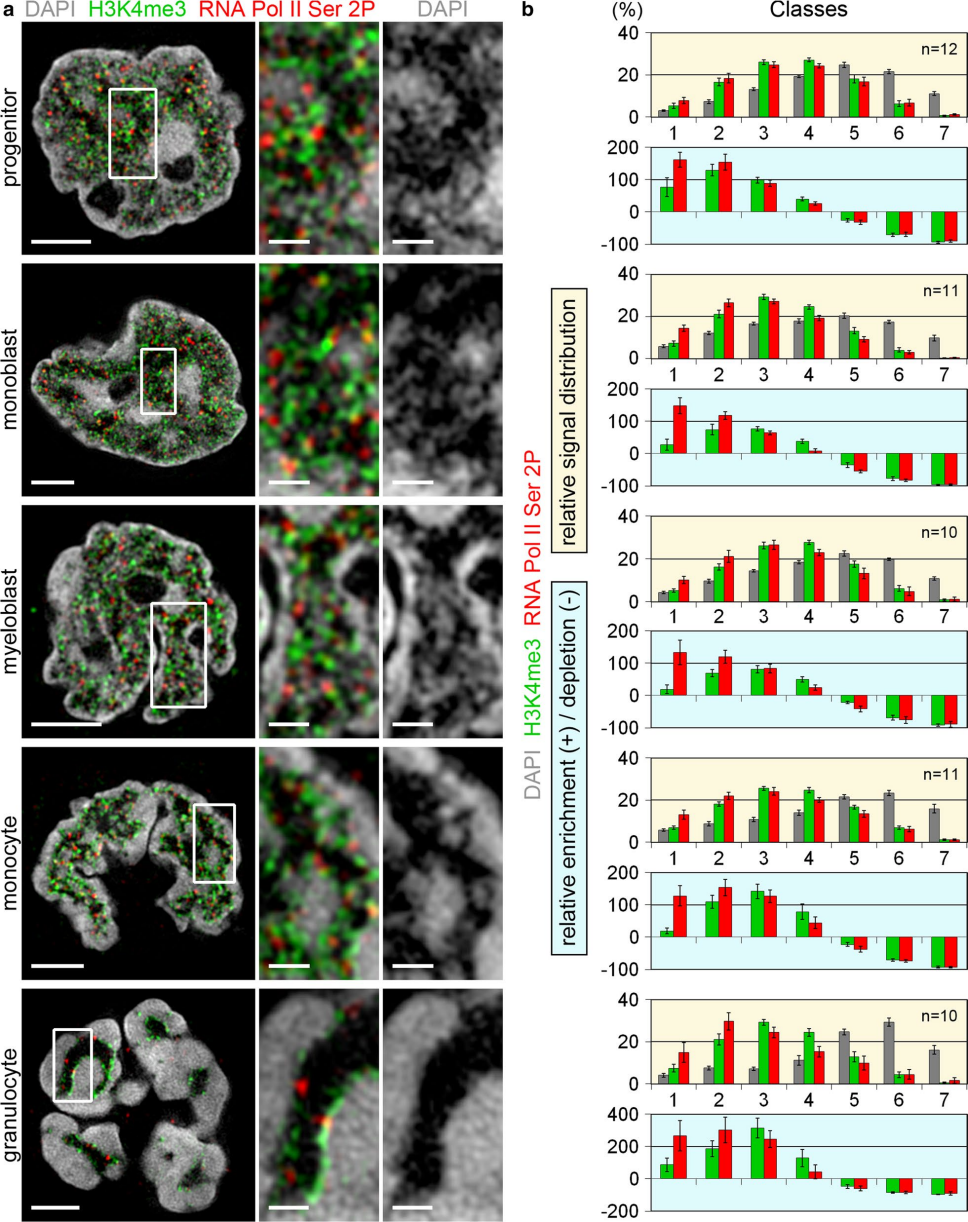
Comparative topology of H3K4me3 and RNA Pol II Ser2P, markers for transcriptionally permissive/active chromatin in relation to chromatin density maps. **a** 3D-SIM light optical mid-sections from 3D acquisitions of whole nuclei and representative inset magnifications delineating DAPI stained DNA (*gray*), immuno-stained H3K4me3 (*green*) and RNA Pol II Ser2P (*red*). All cell types show a preferential localization of H3K4me3 and RNA Pol II Ser2P at decondensed chromatin sites or at the surface of compacted chromatin domain clusters. *Scale bars* 2 µm; *insets* 0.5 µm. **b** *graphs highlighted with yellow background:* relative signal distribution of H3K4me3 (*green*) and RNA Pol II Ser2P (*red*) within respective DAPI defined DNA intensity classes. *p* < 0.001 for DAPI vs. H3K4me3 and RNA Pol II Ser2P in all cell types. *Graphs highlighted with light*-*blue background* quantified levels of relative enrichment (*positive values*) or depletion (*negative values*) of H3K4me3 (*green*) and RNA Pol II Ser2P (*red*) signals relative to the classified DAPI signals. All cell types show a similar profile with a distinct overrepresentation of both markers in low chromatin density classes and a corresponding underrepresentation in high density classes. Note the stronger enrichment of RNA Pol II Ser2P compared to H3K4me3 in class 1 (IC compartment). *n* Number of analyzed nuclei; *error bars* standard deviation

**Fig. 6:**
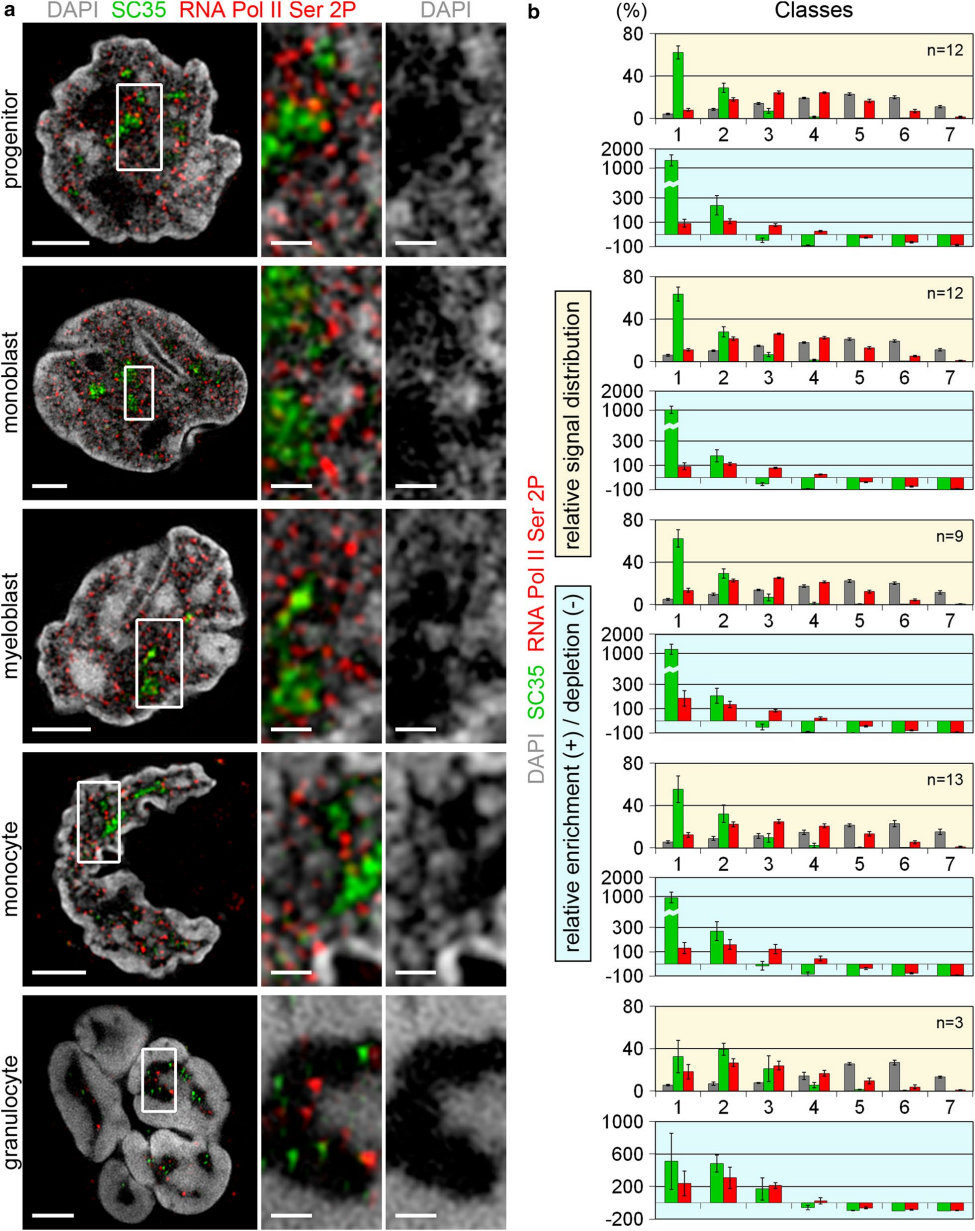
Comparative topology of SC35 and RNA Pol II Ser2P, markers for transcriptional activity in relation to chromatin density maps. **a** 3D-SIM light optical mid-sections from whole 3D acquisitions of nuclei and representative inset magnifications delineating DAPI stained DNA (*gray*), immuno-stained SC35 (*green*) and RNA Pol II Ser2P (*red*). SC35, an integral part of splicing speckles, is seen almost exclusively in the IC compartment, while RNA Pol II Ser2P shows a preferential localization at decondensed chromatin sites or at the surface of compacted chromatin domain clusters (compare Fig. 5). *Scale bars* 2 µm; *insets* 0.5 µm **b** *graphs highlighted with yellow background:* relative signal distribution of SC35 (*green*) and RNA Pol II Ser2P (*red*) within respective DAPI defined DNA intensity classes. *Graphs highlighted with light*-*blue background* quantified levels of relative enrichment (*positive values*) or depletion (*negative values*) of SC35 (*green*) and RNA Pol II Ser2P (*red*) signals relative to the DAPI signals confirm the massive enrichment of SC35 signals in class 1 reflecting the IC compartment. *n* Number of analyzed nuclei; *error bars* standard deviation; *p* < 0.001 for DAPI vs. SC35 and RNA Pol II Ser2P, and for SC35 vs. RNA Pol II Ser 2P

**Fig. 7:**
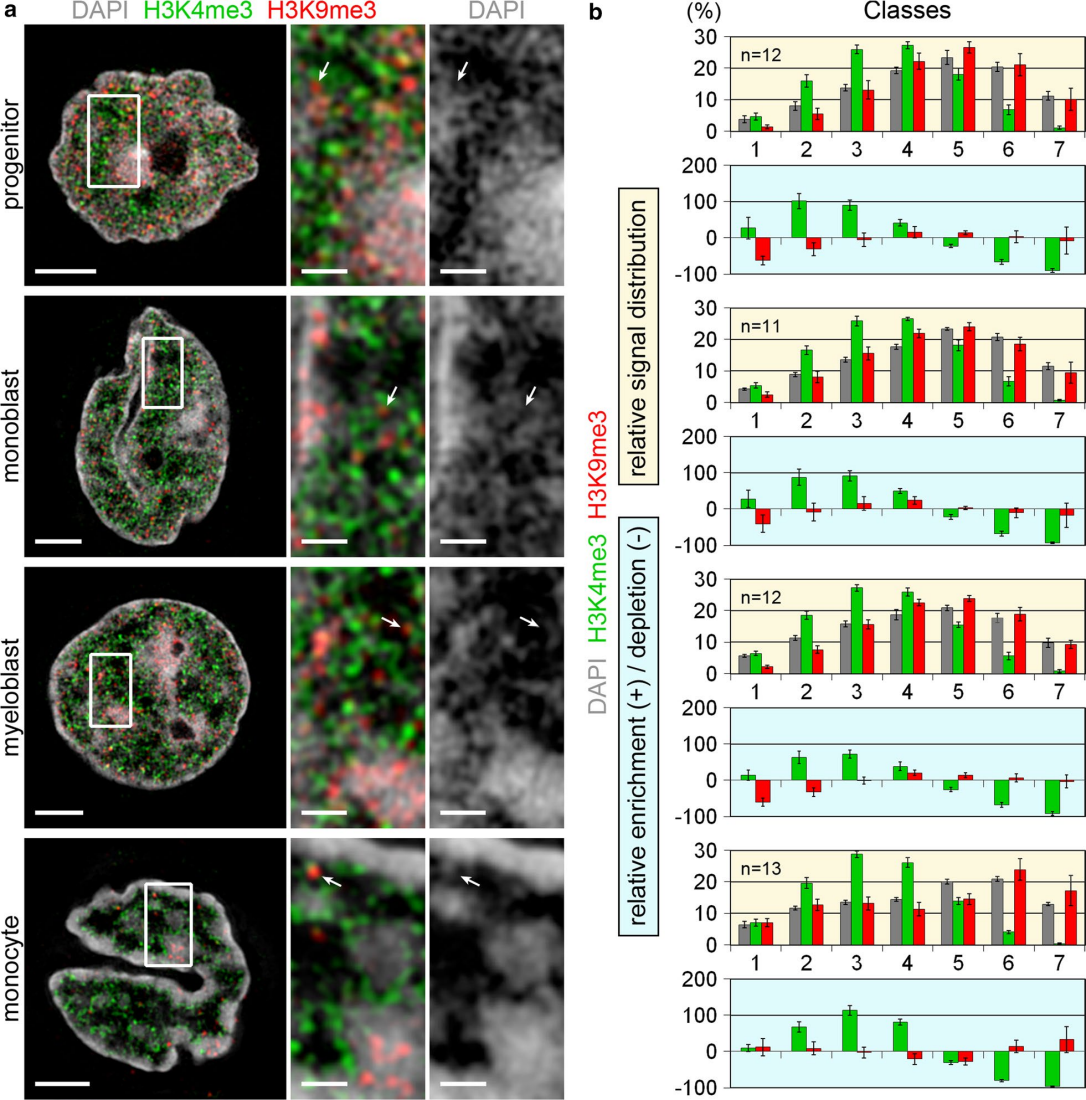
Comparative topology of H3K9me3, a global marker for transcriptionally repressed (hetero)chromatin and H3K4me3 in relation to chromatin density maps. **a** 3D-SIM light optical mid-sections from whole 3D acquisitions of nuclei and representative inset magnifications delineating DAPI stained DNA (*gray*), immuno-stained H3K4me3 (*green*) and H3K9me3 (*red*). H3K4me3 marks decondensed chromatin *sites* and *lines* compacted CDCs (compare Fig. 5). H3K9me3 marks highly compacted chromatin clusters but is also seen at decondensed sites (*arrows*). *Scale bars* 2 µm; *insets* 0.5 µm. **b** *Graphs highlighted with yellow background* relative signal distribution of H3K4me3 (*green*) and H3K9me3 (*red*) within respective DAPI defined DNA intensity classes. *p* < 0.001 for DAPI vs. H3K4me3 and H3K4me3 vs. H3K9me3. *Graphs highlighted with light*-*blue background* quantified levels of relative enrichment (*positive values*) or depletion (*negative values*) of H3K4me3 (*green*) and H3K9me3 (*red*) signals relative to DAPI signals reveal a relative depletion of H3K9me3 signals in classes 1 and 2 in undifferentiated cells (progenitors and precursors) and a relative enrichment of H3K9me3 signals in classes 6 and 7 in monocytes. In both cases the signals are distributed similar to the DAPI intensity classified distributions for the remaining classes. *n* Number of analyzed nuclei; *error bars* standard deviation

Despite their distinctly different nuclear phenotypes on a global level, nuclei from all cell types show a consistent overrepresentation (relative signal enrichment) of the “active” marks H3K4me3, RNA Pol II Ser2P/Ser5P and SC35 in the low-density classes representing the ANC and a corresponding underrepresentation (relative signal depletion) in high density classes considered as the INC (Figs. 5, 6, 7 and Additional files 4, 5). Notably, SC35 is largely restricted to the IC compartment (class 1) (Fig. 6b and Additional file 5B), and RNA Pol II Ser2P/Ser5P signals are both higher enriched in the IC compartment compared to chromatin bound H3K4me3 signals (Fig. 5b and Additional file 4B). The relative signal enrichment/depletion along chromatin density classes for the “silent” mark H3K9me3 (Fig. 7) shows a broader variability even between nuclei of the same cell type (data not shown). On average, in progenitor and precursor cells H3K9me3 signals show the expected underrepresentation (depletion) in classes 1 and 2, while they are fairly proportionally distributed within all other classes. Monocytes show a consistent overrepresentation (enrichment) of H3K9me3 in classes 6–7, while the averaged distribution profile is similar to the DAPI profile in the other classes. Despite these heterogenous results, the relative signal distributions clearly reveal that the majority of the H3K9me3 signals are located in the higher chromatin classes in all cell types. In granulocytes, we failed to detect positive H3K9me3 immunostaining. Failure of H3K9me3 immunostaining in granulocytes was previously described [29, 30] and may be due to a lack or a masking of H3K9me3 epitopes. The access of immunoglobulins required for immunodetection with an estimated size of about 20 nm [31] may in addition be hampered when IC channels fall below a critical throat size of 15–20 nm [32] (an overview on all measured parameters for a comparative topology in relation to chromatin density maps is provided in Additional file 6).

**Fig. 8:**
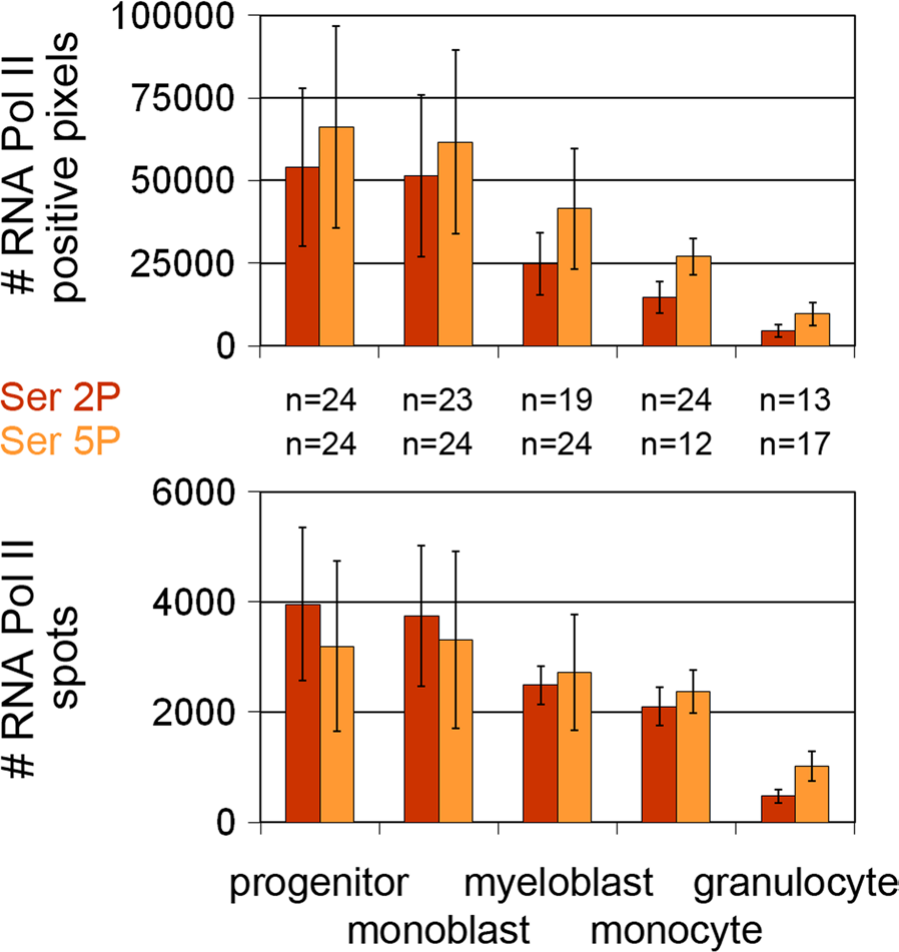
Decrease of RNA Pol II Ser2P/Ser5P signals during myelopoiesis. Quantification of both the number of pixels (*upper graph)* and of spots (see “Methods” part, *lower graph)* reveals a distinct decrease of RNA Pol II Ser2P and Ser5P during differentiation, in particular in granulocytes. *n* number of analyzed nuclei; *error bars* standard deviation

A lowered overall transcriptional activity was previously described for monocytes and resting granulocytes compared to their respective precursor cells [16, 33]. In line with their attenuated transcriptional activity we observed a profound shrinkage of nucleoli in monocytes and granulocytes (Additional file 7) and a decrease of RNA Pol II Ser2P/Ser5P signals defined both by recorded pixels and by custom defined spots (Fig. 8). Recording the number of all signal pixels compensates for differences in size of individually detected spots taking into account that a cell with few large spots can have the same number of positive pixels compared to a cell with many small spots. The counted number of RNA Pol II spots relates to potential “transcription factories”. Current attempts of quantification should be considered with the caveat that the values depend on the threshold setting of recorded images (for a detailed discussion see [6]).

### Transient chromatin decondensation in granulocytic lobes

Living cells are adapted to an osmolarity of about 270 mOsm. Previous studies have demonstrated that a transient incubation of cells in medium with higher or lower osmolarities exerts massive effects on chromatin compaction, which are fully reversible upon restoring normotonic conditions [34]. Hypertonic medium results in increased chromatin compaction, while hypotonic conditions lead to chromatin decondensation [34, 35].

**Fig. 9:**
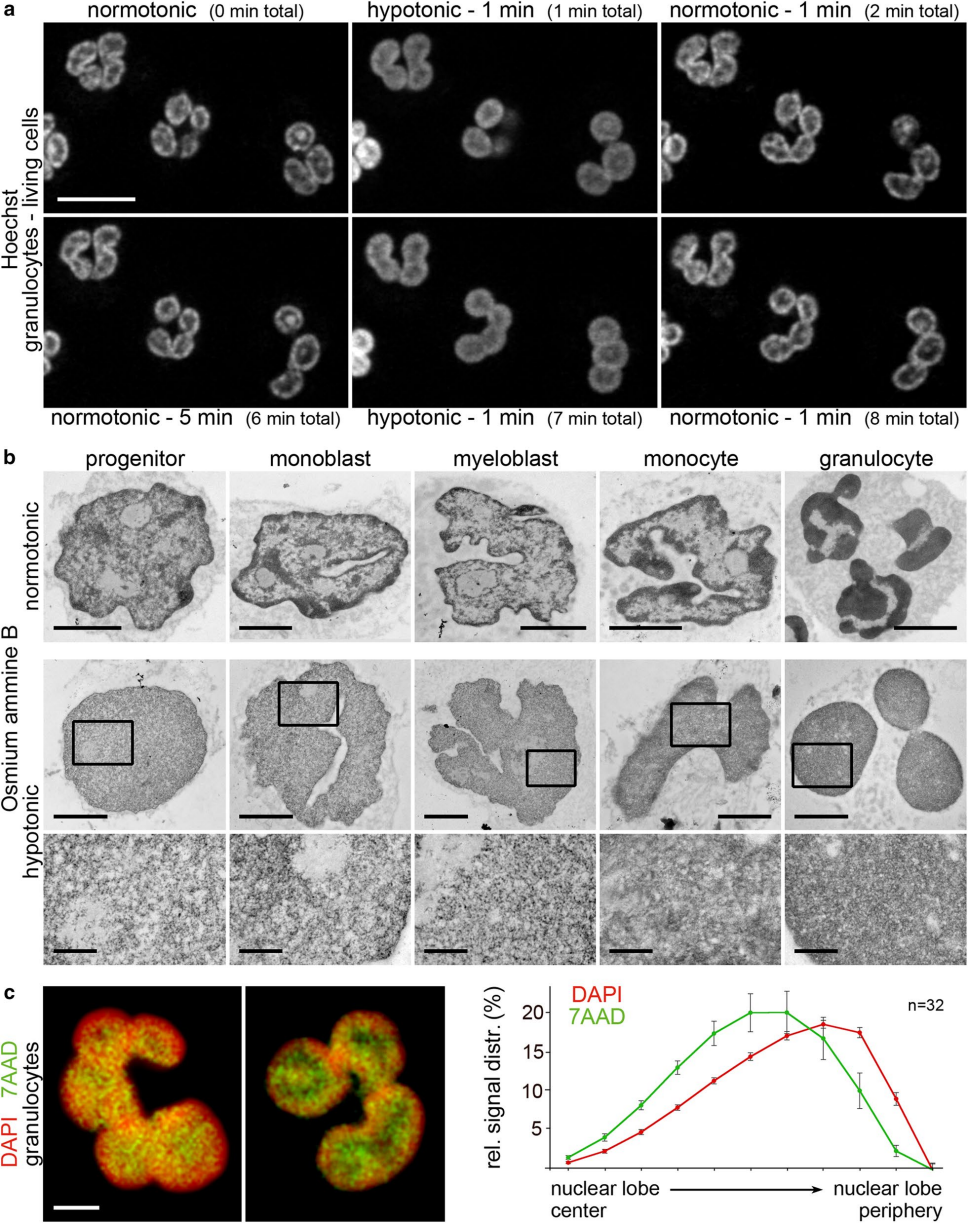
Reversible decondensation of chromatin in myelopoietic cells triggered by hypotonic conditions. **a** Live cell observation of granulocytes with Hoechst33342 stained DNA recorded by spinning disc LSM during repeated circles of normotonic (270 mOsm) and hypotonic (90 mOsm) conditions. The compacted chromatin rim surrounding large interior IC lacunae is seen under normotonic conditions (0 min). Within <1 min in hypotonic conditions a nuclear phenotype appears with (decondensed) chromatin expanding into the IC lacunae. This effect is reversible upon restoring normotonic conditions (2 min) and can be repeated over several cycles (7 and 8 min). *Scale bar* 10 µm. **b** comparison of representative myelopoietic cell nuclei seen under normotonic (*upper panel*) and hypotonic conditions (*lower panel*) in osmium ammine B stained TEM sections. Inset magnifications of hypotonic TEM sections demonstrate a similar, rather even distribution of (decondensed) chromatin throughout the nucleus in all cell types, with loss of larger IC channels and IC lacunae, in particular evident in monocytes and granulocytes. *Scale bars* 2 µm; *insets* 0.5 µm. **c** *left panel:* Different staining intensities after simultaneous DNA staining of granulocytes fixed after 30 s incubation in hypotonic conditions with DAPI (*red*) and 7-AAD (*green*). 7-AAD (high affinity to GC-rich regions) denotes the lobe interior while DAPI (high affinity to AT-rich regions) strongly stains the peripheral rim. This radial divergence of the two dyes illustrates a preferential expansion of GC enriched (gene dense) DNA segments toward the nuclear interior. *z*-projections of 400 nm axial distance are shown. *Right panel* 3D distance measurements of DAPI (*red*) and 7-AAD (*green*) signals to the nuclear border of granulocyte lobes confirm their significantly distinct radial distribution (*p* < 0.001, assessed by Mann–Whitney rank sum test). The ordinate denotes the normalized sum of voxel intensities for a respective fluorochrome, the abscissa the relative distance to the nuclear border. *n* number of analyzed nuclei; *error bars* standard error of means

The huge IC lacunae of granulocytes observed under normotonic conditions may serve as a storage compartment and be tightly filled by macromolecular complexes and nuclear bodies, whose nature was not analyzed in the present investigation. In a live cell experiment, we tested effects of chromatin decondensation triggered by hypotonic conditions on the appearance of IC lacunae in granulocytes by exposing cells to a transient change from normotonic to hypotonic medium (~90 mOsm). Within <1 min in hypotonic medium chromatin expands into the large IC lacunae of granulocytic lobes and fills their space, resulting in a wide dissolution of the highly compacted, distinct peripheral chromatin layer typically seen in these lobes under normotonic conditions. This state can be rapidly restored (<1 min) after reincubation in normotonic medium. Repeated cycles of chromatin decondensation and compaction in granulocytes upon transient exposure to hypotonic and normotonic medium were observed (Fig. 9a). Osmium ammine B stained TEM sections of progenitors, precursors, monocytes and granulocytes, kept in hypotonic medium (~90 mOsm) for 1 min before fixation, demonstrate that hypotonic conditions trigger a rather uniform distribution of decondensed chromatin throughout the nuclear space in all cell types (Fig. 9b). These observations argue against a crowding of space-occupying nuclear bodies within the central IC lacunae of lobes and provide indirect evidence for a higher order chromatin organization, which allows a rapid, transient chromatin de- and re-condensation (see “Discussion”).

Co-staining of granulocytes with DAPI (high affinity to AT-rich regions) and 7-AAD (high affinity to GC rich regions) that were fixed after 30 s incubation in hypotonic conditions reveals remarkable differences of the staining patterns. 7-AAD denotes the lobe interior, while DAPI strongly stains the peripheral rim (Fig. 9c). This radial polarization of the two dyes illustrates a preferential expansion of GC enriched (gene dense) DNA segments toward the nuclear interior. This GC enriched DNA likely represents the transcriptionally competent chromatin fraction residing as ANC at the interface between the compact, peripheral chromatin layer and the internal lacunae in granulocytic lobes.

### Radial arrangement of gene-dense and gene-poor chromatin is maintained in granulocytic lobes

**Fig. 10:**
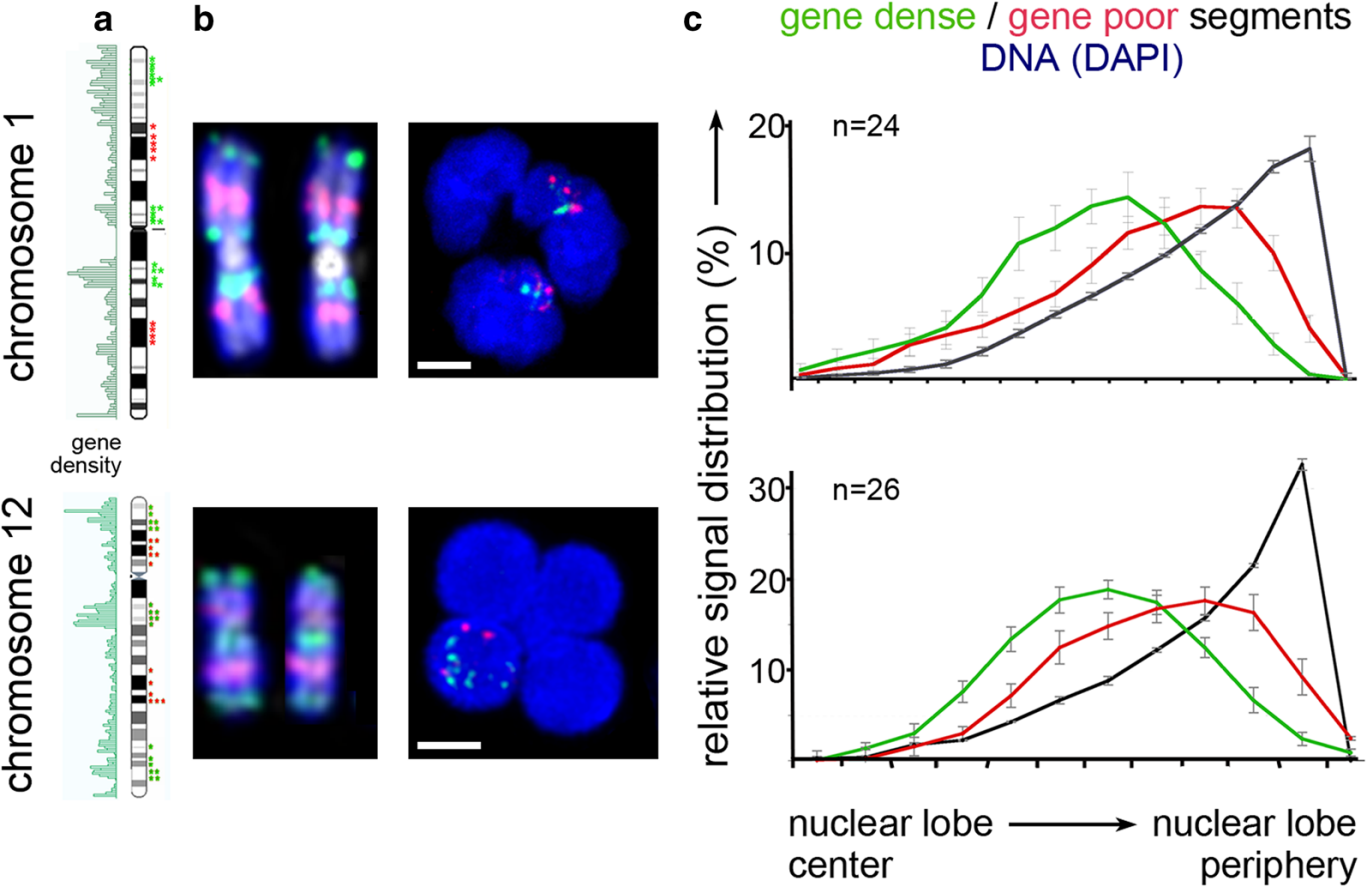
Distinct radial positioning of gene-poor and gene-dense segments of chromosomes 1 and 12 with regard to the border of granulocyte nuclear lobes. **a** ideograms of chromosomes 1 and 12 with regional gene density (*left bar*). Localization of individual BAC clones representing either gene-dense (*green*) or gene-poor segments (*red*) used in this study is marked by an *asterisk*. **b** *left panel*: respective 2D-FISH control experiments with the expected banded pattern on metaphase chromosomes; *right panel:* *z*-projections of 3D-FISH experiments. **c** 3D measurements for the distance distributions of signals delineating gene-dense (*green*) and gene-poor (*red*) segments, respectively, reveal their significantly distinct radial distribution both for chromosome 1 (*top*) and for chromosome 12 (*bottom*); (p < 0.005 for both *curves*, assessed by Mann–Whitney rank sum test). The ordinate denotes the normalized sum of voxel intensities for a respective fluorochrome, the abscissa the relative distance to the nuclear border. Nuclear counterstain (DAPI) is denoted in *blue*. *n* number of analyzed nuclei; *error bars* standard error of means

A radial gene density correlated arrangement of chromatin segments with the preferential localization of gene-poor chromatin at the nuclear periphery and gene-dense chromatin toward the nuclear interior has previously been described in several studies and was consistently confirmed for a large number of cell types and species (for review see [36]). For a further comparison of the topography of gene-dense and gene-poor chromatin regions in granulocytic lobes, we performed 3D-fluorescence in situ hybridization (3D-FISH) with differentially labeled sets of pooled BAC clones from human chromosomes HSA #1 and HSA #12 delineating gene-dense and gene-poor segments of these chromosomes (previously described in [37, 38]). We selected HSA #1 and #12 since they represent chromosomes with an overall intermediate gene density (19 and 18 genes/Mb, respectively, http://www.ncbi.nlm.nih.gov/genome/guide/human/), however, with distinct segmental differences of regional gene density within size windows of several Mbs (Fig. 10a, b). Confocal image stacks, recorded from granulocyte nuclei, were used for 3D distance measurements of the respective probe signals obtained with regard to the DAPI delineated border of a granulocytic nuclear lobe. The results demonstrate a gene density correlated radial arrangement with gene-dense chromatin oriented toward the IC lacuna and gene-poor chromatin enriched toward the nuclear border (Fig. 10c).

The maintenance of the conventional gene density correlated chromatin arrangement in multilobulated nuclei of granulocytes further confirms the general validity of such a radial arrangement. The only reported exception of an inverted architecture so far is rod cell nuclei of nocturnal mammals, where these cells act as micro-lenses for effective light transmission [39].

## Discussion

### Local similarities of nuclear organization despite major global differences of nuclear architectures in human myelopoietic cell types

3D structured illumination microscopy (3D SIM), complemented by transmission electron microscopy (TEM), was used for a comparative study of nuclear landscapes during myelopoietic cell differentiation, including CD34+ progenitor cells, myeloid precursors (monoblasts and myeloblasts) and mature monocytes and granulocytes. Nuclei of all cell types carry a higher order chromatin network, composed of chromatin domain clusters (CDCs) with a compact core and a less condensed chromatin periphery, the perichromatin region (PR). The entire chromatin network is permeated by a co-aligned network of IC channels and occasional larger IC lacunae. IC channels are connected to nuclear pore complexes (NPCs). The topography of higher order chromatin arrangements and the nearly DNA-free interchromatin compartment (IC), however, differs starkly in different cell types. In CD34+ progenitor and precursor cells, this topography resembles the pattern previously observed by 3D SIM in various somatic cell lines [2, 5, 35]. In contrast, monocytes show larger aggregates of closely packed CDC islets, surrounded by wide IC channels, and multilobulated nuclei of granulocytes are characterized by a compact peripheral chromatin layer around a large internal IC lacuna, from which sparse, narrow channels penetrate the compact peripheral chromatin layer toward comparatively infrequent NPCs. The peripheral layer of granulocytic lobes likely represents a higher order chromatin organization of densely arranged CDCs with a strongly collapsed IC channel system, but other more intertwined chromatin arrangements have to be considered. Despite these major differences between nuclear landscapes of different myelopoietic cell types at a global scale, we noted a consistent organization between all cell types at a more local scale with regard to a functional compartmentalization into an active and an inactive nuclear compartment (ANC and INC). This consistency is most evident for the ANC, exemplified in our study by its relative enrichment with H3K4me3, a histone mark indicating transcriptional competence, RNA Pol II Ser2P/Ser5P representing initiating and elongating forms of active RNA Pol II [28] and SC35, a protein involved in transcriptional elongation [27] and RNA splicing [40]. SC35 signals are strongly enriched within the largely DNA-free IC, whereas H3K4me3 is mostly overrepresented within the decondensed chromatin layer which forms the interface between the core of CDCs and the IC. RNA Pol II signals show an intermediate localization, with enrichments both within the IC and the PR.

H3K9me3 was employed as a marker for silent chromatin and compared to the “active” marks shows a higher variability between and even within cell types. Nuclei of progenitors and precursors, but not of monocytes, show on average the expected underrepresentation of H3K9me3 in the lowest DAPI intensity classes while this epigenetic mark largely reflects the DAPI distribution profile in the higher classes. Notably, H3K9me3 is involved in fine tuning of expression levels at promoters and enhancers for large-scale repression [25].

Differences in the topography of epigenetic markers and other proteins between individual cells and cell populations, respectively, may reflect a stochastic cell-to-cell variability, functional differences between cells at different stages of differentiation or during the cell cycle, as well as technical issues. The classification of DAPI intensity classes and the assignment of signal pixels from immuno-stained markers depends on parameters such as the chosen threshold. Cell-to-cell variability of staining efficiency has also to be taken into account as a caveat against an overinterpretation of differences detected even between nuclei from cell neighbors and in the interpretation of differences obtained from different experiments. However, we wish to emphasize the high reproducibility of the major result, which has emerged from our current approach of a quantitative assessment of the nuclear topography of chromatin and functional markers. The whole set of data so far obtained with this approach, including nuclei from mouse, human and bovine somatic cells, mouse embryonic stem cells and bovine preimplantation embryos obtained by in vitro fertilization and somatic cell nuclear transfer [2, 3, 5, 6] and our present study, consistently demonstrates an enrichment of H3K4me3, RNA Pol II Ser2P/5P and SC35 in DAPI intensity classes attributed to the ANC. An enrichment of epigenetic markers assigned to transcriptionally competent chromatin was also observed in a most recent study of nuclei from the mouse cardiomyocyte cell-line HL-1 performed with super-resolved, single-molecule localisation microscopy of DNA binding dyes together with immunodetection of H3K14ac [41]. The fact that a highly non-random enrichment of such epigenetic markers with the PR was detected by super-resolved microscopy based on different physical principles rules out that our findings may result from artifacts of the complex algorithms involved in 3D-SIM generated images.

We still lack information on the possible redistribution of functionally relevant DNA between the ANC and INC during cell differentiation from precursors to (terminally) differentiated cells. We have raised this important problem in a recent review [7]: “With respect to the topography of regulatory and coding sequences we consider several scenarios: (a) regulatory sequences of genes may be exposed within the ANC independent of whether genes are active or inactive. (b) Only regulatory sequences, which are in active use in a given cell, may be located within the ANC, whereas regulatory sequences of genes shut off in this cell are retracted into the INC represented by the compact and largely inaccessible interior of CDCs. In this case shifts of regulatory sequences embedded within the INC into the ANC should occur prior to the activation of the respective genes, while repositioning of such sequences from the ANC into the INC should occur, when these genes become inactive. (c) Similar considerations apply to coding sequences”. It should be noted that nascent RNA was shown to be preferentially synthesized in a narrow layer of decondensed chromatin, called the perichromatin region located at the periphery of chromatin domain clusters (for review see [42]). Accordingly, a potential repositioning of genes between the ANC and INC may typically take place at scales below 200 nm (see also [7]). Experimental studies to solve these issues pose an enormous challenge. Multicolor 3D-FISH experiments combined with super-resolution fluorescence microscopy can be considered, yet such experiments bear the danger that destruction of fine details of chromatin structure prohibit a clear answer at this size scale [3]. Recently, it has become possible to visualize specific DNA sequences in nuclei of living cells [43]. Yet, despite their enormous promise, these new technologies have not reached the state of routine applications.

### Nuclear organization and function in myeloid cells

Monocytes and granulocytes have an overall reduced transcriptional activity of a large number of genes compared to their precursors [16], in our study reflected by the relative paucity of active RNA Pol II signals and tiny nucleoli. Both, monocytes and in particular granulocytes are, however, capable of a rapid transcriptional upregulation of numerous specific genes upon environmental stimulation, such as exposure to bacterial lipopolysaccharides or lectin stimulation [16, 17, 44–46]. It may be speculated that an architectural configuration with a diminished surface area of the interface between decondensed chromatin and the IC, as it is established in nuclear lobes of mature granulocytes and in monocytes, reduces the dwell time of transcription factors in search for their specific targets [32] and may speed up regulation of genes.

Monocytes and granulocytes can migrate into tissues to the site of bacterial invasion within minutes by squeezing through the tight endothelial wall of blood vessels. Efficiently squeezing through the tight endothelial wall of blood vessels requires a high deformability of nuclei. Cytoskeletal filaments, in particular actin [47] and the specific composition of the nuclear envelope have been found important for nuclear shape determination and deformability in human granulocytes and monocytes [45, 48]. The particular chromatin organization in these cell types may favor a high malleability of nuclear shapes. The large internal IC lacunae in granulocyte lobes are apparently not packed with larger, space filling and rather insolvable complexes, such as nuclear bodies. This was shown by incubation of living human granulocytes in hypotonic medium resulting in a rapid shift of chromatin from the peripheral chromatin layer into the IC lacunae. This effect was fully reversible, when the cells were again incubated in normotonic medium. While we lack evidence for the actual organization of compacted chromatin in the periphery of granulocytic lobes, these observations provide indirect evidence for a higher order chromatin organization, which allows a rapid, transient chromatin de- and re-condensation. It has been speculated that the possibility of a rapid expansion and re-compaction of chromatin domains plays a major role in gene regulation [49]. In line with this assumption, it has been argued that chromatin domains may be built up from smaller globules like Russian matryoshka dolls [50, 51]. It is interesting to note that chromatin domains can be modeled as a polymer analog of a 3D Peano curve [first described by Guiseppe Peano (1858–1932)] [52] which fill higher dimensional space as entirely unknotted structures. Such an organization prevents chromatin entanglements during the de- and re-condensation of chromatin domains. Direct evidence for such an organization is lacking and currently very difficult to obtain [53].

The ANC-INC network model argues that the IC channel system serves as a system for nuclear import–export functions [23] (for review see [7]). This hypothesis is in part based on a study, which reported that interchromatin channels in mammalian cell nuclei ensure “a steady and continuous wave of mRNPs traveling toward the nuclear pore complex (NPC)” [54]. It implies the assumption that the compact core of chromatin domain clusters is much less accessible for both RNPs produced within the ANC as well as for factors which enter the nucleus through NPCs to form aggregates involved in transcription, splicing, DNA replication and repair preferentially within the ANC. Multi-scale fluorescence cross-correlation spectroscopy analysis of the mobility of inert monomers, trimers and pentamers of GFP, as well as GFP fusions with other proteins in nuclei of living cells [32] indicated that the nuclear interior acts like a sponge like structure. Chromatin domains may act as obstacles for individual factors and aggregations. In this type of nuclear organization, the protein–chromatin interactions at the obstacle surface occur in a particle-size dependent manner. Accordingly, the decondensed chromatin surface surrounding lacunas may become more easily accessible for functional macromolecules and macromolecule complexes than the much more compacted interior of CDCs. In a most recent study multifocus microscopy (MFM) was carried out to capture 3D single-molecule real-time images in living cells [55]. It was concluded that β-actin mRNAs freely access the entire nucleus. The limited resolution imposed by conventional light microscopy employed in this study, however, does not exclude a diffusion along IC channels. Further high-resolution microscopic studies, e.g. by super-resolved MFM, are necessary to decide whether RNPs can take any routes throughout the peripheral chromatin layer for their passage toward nuclear pores or whether this passage occurs along IC channels that end directly at nuclear pores. Human granulocytes may serve as a useful model system for such studies assessing the potential importance of IC channels for nuclear import and export functions since their peripheral chromatin layer is pervaded by few and narrow IC channels. This topography provides good opportunities to decide whether mRNAs and RNPs, respectively, can take the entire layer of peripheral chromatin for their passage toward NPCs or whether this passage occurs preferentially along such narrow IC channels.

Conventional TEM sections suggest that the interior of chromatin domain clusters is not solid as suggested by the liquid drop model, but composed of fibrous chromatin arrangements, which arguably provide sufficient space for RNP export through such clusters [35]. For a long time, it has been argued that both 10-nm beads-on-a string chromatin fibers and 30 nm thick higher order chromatin fibers exist in vivo [56]. Despite undisputable evidence for the formation of 30 nm chromatin fibers in vitro [57], studies based on cryo-EM and X-ray scattering have argued that the interior of chromatin domains is so densely packed that nucleosome interactions between 10 nm thick chromatin fibers prevent the formation of 30 nm thick fibers [58–60]. To what extent 30 nm chromatin fibers may play a role in chromatin compaction in vivo is still an unsettled question. These problems sound a note of caution against naïve biological interpretations of images without a careful consideration of potential artifacts. A safeguard against misleading interpretations is the application of a set of different methods including novel correlative fluorescence—electron-microscopic (CFEM) approaches, as well as live cell studies.

## Conclusions

This study demonstrates consistent hallmarks of nuclear landscapes present in all cell types of the human myelopoietic lineage irrespective of major differences of global chromatin arrangements. These hallmarks include the presence of co-aligned networks of an active (ANC) and an inactive (INC) nuclear compartment. The ANC starts with interchromatin compartment (IC) channels connected to nuclear pores and pervades the nuclear interior together with its co-aligned INC. ANC and INC differ with regard to functionally relevant hallmarks, including a highly significant enrichment of RNA polymerase II and histone signatures for transcriptionally competent chromatin in the ANC, whereas the INC is enriched in marks for repressed chromatin. These results are in line with the functional organization of nuclei previously observed in a variety of mammalian species, including bovine blastomeres, mouse embryonic stem cells, and various somatic cells, reviewed in [7]. We conclude that the ANC-INC network model of nuclear organization reveals evolutionary conserved principles of the functional nuclear organization. To which extent these principles may be valid for eukaryote species in general remains to be seen.

## Methods

### Isolation of hematopoietic cells of defined differentiation stages

Human CD34+ cells (progenitors) were purified from umbilical cord blood (CB) samples by magnetic cell sorting. Samples were obtained from the Emilia Romagna Cord Blood Bank (ERCB) at the policlinic S. Orsola-Malpighi in Bologna. Myeloid precursors (monoblasts and myeloblasts) were obtained by in vitro differentiation of CB derived CD34+ cells as described in [15]. Separation of monoblasts and myeloblasts was achieved by selection for the surface antigen CD14: monoblasts are CD14+, myeloblasts are CD14−. Monocytes were isolated from peripheral blood using either magnetic cell sorting or Histopaque gradients. Granulocytes were isolated from peripheral blood using Histopaque gradients (for a detailed description see Additional file 8).

### Immunodetection

Antibodies used for immunodetection: primary antibodies against RNA Pol II Ser2P/Ser5P (rat monoclonal; kindly provided by D. Eick LMU Munich), H3K4me3 (rabbit polyclonal; Abcam, ab8580), H3K9me3 (mouse monoclonal; Active Motif, 39285), SC35 (mouse monoclonal; Sigma-Aldrich, S4045), nucleoli (human nucleolar positive control; Antibodies Incorporated) and secondary antibodies against rabbit coupled with DyLight488 (raised in donkey; Jackson Immuno Research), against mouse and rat coupled with Alexa594 (raised in goat, respectively donkey; Molecular Probes) and against human coupled with FITC or Alexa594 (raised in goat; Jackson Immuno Research or Molecular Probes, respectively). A detailed protocol for fixation and immunodetection procedure meeting the requirements for 3D-SIM imaging is provided in Additional file 8.

### 3D-SIM

Super-resolution structured illumination imaging was performed on a DeltaVision OMX V3 system (Applied Precision Imaging/GE Healthcare) equipped with a 100×/1.4 UPlan S Apo oil immersion objective (Olympus), Cascade II:512 EMCCD cameras (Photometrics) and 405, 488 and 593 nm lasers [61]. Raw data image stacks were acquired with 15 raw images per plane (5 phases, 3 angles) and an axial distance of 125 nm and then computationally reconstructed with a Wiener filter setting of 0.002 and channel specific optical transfer functions (OTFs) using SoftWoRx (Applied Precision) [24, 62]. The reconstruction process generates 32-bit data sets with the pixel number doubled in the lateral axes, resulting in the pixel size being halved from 79 to 39.5 nm to meet the Nyquist sampling criterion. The level of spherical aberration was minimized and matched to the respective OTFs using immersion oil of different refractive indices (RI). Best results were typically obtained with OTFs measured on red, green (both 110 nm diameter) and blue (170 nm diameter) fluorescent FluoSpheres (Invitrogen) using RI 1.512, and sample acquisition with RI 1.514 for depth adjustment in the region of optimal reconstruction a few µm into the sample. Images from the different color channels were corrected for chromatic aberration in SoftWoRx with alignment parameters obtained from calibration measurements with 0.2 µm diameter TetraSpeck beads (Invitrogen). If necessary (e.g. for large cells in axial direction) the alignment parameters were manually adjusted to compensate for the larger distance from the coverslip. To normalize all image stacks for subsequent image processing and data analysis, the original 32-bit images were shifted to positive values and transformed to 16-bit. Then the stack specific mode gray value (representing the peak of the background noise) was subtracted, unless the images were used for chromatin density classification described below. All further image processing was done in ImageJ (http://rsb.info.nih.gov/ij/). 3D models of image stacks were generated in Amira (FEI Visualization Sciences Group).

### Chromatin density classification by 3D assessment of DAPI intensity classes

For chromatin density quantification signals of DAPI stained DNA were segmented into seven classes with equal intensity variance using an in-house algorithm described in [5, 6]. A hidden Markov random field model classification was used, combining a finite Gaussian mixture model with a spatial model (Potts model), implemented into the statistics software R [63, 64].This approach allows threshold-independent signal intensity classification at the voxel level, not only based on the intensity of an individual voxel but also considering the classification of surrounding voxels. In our study the influence of the neighboring voxels was set to 0 as this yielded a better correspondence of the segmented images with the original images. Prior to chromatin density classification 3D nuclear masks were generated using the same segmentation algorithm, but without implementing a mask, followed by setting an appropriate threshold in ImageJ and if necessary further processing using dilate/erode functions and manual corrections. Care was taken that invaginations of the nuclear surface were maintained in the mask. All signals outside the nucleus were deleted from the mask. For the allocation of immunodetected marker signals (H3K4me3, RNA Pol II Ser 2P/Ser5P, SC35, H3K9me3) in relation to chromatin density the marker channels were thresholded and the individual voxels correlated with the corresponding voxel in the DAPI channel and assigned to the respective DNA intensity class. Statistical significance was tested by a Wilcoxon rank sum test with continuity correction. Over-/underrepresentations (relative depletion/enrichment) of the marker signals were calculated by setting the difference between the relative amount of signals in the marker channel and the corresponding DAPI channel in relation to the relative amount of signals in the DAPI channel.

### Quantification of RNA Polymerase II positive pixels or spots

The number of RNA Polymerase II positive pixels was summed up from the number of pixels in the seven density classes obtained in the density classification described above. The number of spots within the segmented nuclear mask was determined in Volocity (PerkinElmer) using the “find spots” function: an appropriate offset for spot intensity was chosen in “extended focus” view and the minimum distance between spots was set to 0 µm. The total number of summed up pixels per cell and the number of “spots” were tested for statistical significance between cell types using a Wilcoxon rank sum test with continuity correction.

### Sample preparation for TEM and osmium ammine B staining

Fixation of cells under normotonic conditions was performed with 4 % paraformaldehyde/1× PBS for 10 min, fixation under hypotonic conditions was performed by incubating the cells in 0.3× PBS for 1 min prior to fixation with 4 % paraformaldehyde/0.3× PBS for 10 min, followed in both cases by washing with 1× PBS. Further preparation of samples and cutting of ultrathin sections (100 nm) was essentially performed as described in [35]. Staining of DNA in 2.8 mM osmium ammine B was essentially done according to [65]. For a detailed description of TEM sample preparation and osmium ammine B staining see Additional file 8. Ultrathin sections were imaged on a FEI Morgani 268 operated at 80 kV. 8-bit or 16-bit gray scale 2D images were acquired at several magnifications with an average gray value of 50–60 %, taking care not to cut off signals on both sides of the spectrum (high and low values). The alignment of different magnifications of each nucleus to each other was performed in Adobe Photoshop using the rotate, resize, warp and distort functions. Further image processing was done in ImageJ.

### Quantification of the chromatin/IC interface length in TEM sections

Nuclear masks of osmium ammine B stained nuclei were generated as described above for the masks used for the chromatin density classification of 3D-SIM images. The IC/chromatin interface length was calculated by subtracting the perimeter of the nucleus (determined from the mask in Volocity, (PerkinElmer) from the perimeter of the chromatin threshold, set on the corresponding original image in Volocity. The results were normalized for the area of the nuclei. The Wilcoxon rank sum test with continuity correction was used for testing for statistical significance.

### Live cell observation of granulocytes

Live cell observations of granulocytes during changes between normotonic (live cell medium) and hypotonic conditions (0.3× PBS) were performed on an Axio Observer D1 (Zeiss) with an UltraView VoX spinning disk unit (PerkinElmer) and an 63×/1.4 plan-apochromat oil objective. Cells seeded into glass bottom dishes (MatTek) as described above for immunodetection were stained with 0.3 µg/ml Hoechst 33342 in live cell medium (DMEM without phenol red (Invitrogen), 50 mM Hepes, 10 % FCS) for 30–45 min. After replacing the medium with fresh live cell medium, stacks of 8-bit gray scale images were acquired with an axial distance of 300 nm between optical sections using the 405 nm laser line for excitation and FITC filter settings for emission detection. After completion of image acquisition the medium was exchanged with 0.3× PBS and image stacks were acquired again, resulting in an incubation time of ~1 min in 0.3× PBS. Subsequently the 0.3× PBS was changed back to live cell medium and image stacks were captured again. Another image stack was acquired after an additional 3 min incubation (i.e. total incubation in medium: 5 min) before repeating the cycle.

### 3D-FISH and 3D distance measurement of fluorescence signals to the nuclear border

Pooled BAC probes, assigned to either gene-dense or gene-poor segments of human chromosomes 1 and 12 (19 clones for gene-dense segments of chr. 1 and of chr. 12, 9 and 12 clones, respectively, for gene-poor segments; compare Fig. 10), were differently labeled with haptens and used for the delineation of the respective sequences in human granulocytes. Probe setup, fixation in 4 % PFA/0.5 × PBS, pretreatment of cells, hybridization, DAPI staining and detection by Cy3- or Cy5-conjugated antibodies were performed as previously described [38, 66]. Nuclei were scanned using a laser scanning confocal microscope (Leica SP5) equipped with a 63×/1.4 plan-apochromat oil objective. Stacks of 8-bit gray scale 2D images collected sequentially for all fluorochromes were obtained with a pixel size of 50 nm and an axial distance of 200 nm between optical sections. Images were processed with ImageJ. Chromatic aberration was corrected with alignment parameters obtained from the measurement of multi-colored fluorescent beads and an in-house plugin for ImageJ. For the measurement of the shortest absolute 3D distances of all BAC signals to the surface of the segmented nuclear border, an in-house software (“enhanced absolute 3D distances to surfaces,” eADS) was used, previously described in detail in [38]. For a detailed description see Additional file 8.

### Simultaneous DAPI and 7AAD staining

Granulocytes on coverslips were incubated in 0.5× PBS for 30 s and then fixed with 4 % paraformaldehyde/0.5× PBS for 10 min. The cells were stained with DAPI and 7AAD and images were acquired, processed and evaluated as described above for 3D-FISH.

### Ethics statement

Human CD34+ cells were purified upon donor’s informed written consent from umbilical cord blood (CB) samples, collected after normal deliveries, according to the institutional guidelines for discarded material (Clearance of Ethical Committee for Human experimentation of Modena: Secretary office Saverio Santachiara, santachiara.saverio@policlinico.mo.it, approval date: 18.01.2005; approval file number # 793/CE).

## Additional files

10.1186/s13072-015-0038-0 Exit points of IC channels at the nuclear surface in myelopoietic cell nuclei. Exit points of IC channels connected to nuclear pores were previously shown to appear as little holes in the nuclear envelope [5,7,23,24]. 3D reconstructions using Amira software (compare Fig. 2B) of whole 3D-SIM 3D acquisitions of DAPI stained nuclei from progenitors, precursors, monocytes and granulocytes were used for a quantitation of such exit points. *Upper graph:* Number per nucleus; *lower graph:* average nuclear surface area harboring one exit point. Number of exit points is significantly decreased in monocytes and granulocytes compared to their respective precursors (p < 0.001). Displaying the results as nuclear surface area harboring one channel exit demonstrates the profound difference between granulocytes and the other four cell types (p ≤ 0.001). n = number of analyzed nuclei; error bars = standard deviation.
10.1186/s13072-015-0038-0 Section galleries of nuclei shown in Fig. 2. Galleries of light optical serial sections (axial distance = 125 nm between each optical section) of whole 3D-SIM 3D acquisitions of the DAPI stained nuclei shown in Fig. 2. For the progenitor cell every fifth image (axial distance = 625 nm), for the monoblast every second image (axial distance = 250 nm), for myeloblasts, monocytes and granulocytes every third image (axial distance = 375 nm) is included. Nuclei of progenitors exhibit an overall roundish shape with invaginations at the surface. Monoblast nuclei are of ellipsoid shape with typically deep and complex invaginations. Nuclei of myeloblasts are similar to monoblast nuclei; however, typically they are slightly thicker, and invaginations often pervade the whole nucleus. Monocytes are characterized by horseshoe-shaped nuclei with an irregular surface. Nuclei of granulocytes are divided into several interconnected lobes.
10.1186/s13072-015-0038-0 DAPI intensity classification profiles from individual nuclei. Four representative chromatin density profiles based on seven DAPI intensity classes are shown for each cell type demonstrating the similarity of profiles within a given cell type and the overall shift towards higher intensity classes in differentiated cell types (monocytes and granulocytes). For comparison the average profiles are repeated from Fig. 4.
10.1186/s13072-015-0038-0 Comparative topology of H3K4me3 and RNA Pol II Ser5P, markers for transcriptionally permissive/active chromatin in relation to chromatin density maps. **(A)** 3D-SIM light optical mid-sections from whole 3D acquisitions of nuclei and representative inset magnifications delineating DAPI stained DNA (gray), immuno-stained H3K4me3 (green) and RNA Pol II Ser5P (red). All cell types show a preferential localization of H3K4me3 and RNA Pol II Ser5P at decondensed chromatin sites or at the surface of compacted chromatin domain clusters. Scale bars: 2 µm; insets 0.5 µm. **(B)** *graphs highlighted with yellow background:* relative signal distribution of H3K4me3 (green) and RNA Pol II Ser5P (red) within respective DAPI defined DNA intensity classes. p < 0.005, except for H3K4me3 vs. RNA Pol II Ser5P in progenitors (p = 0.059). *Graphs highlighted with light*-*blue background:* quantified levels of relative enrichment (positive values) or depletion (negative values) of H3K4me3 (green) and RNA Pol II Ser5P (red) signals relative to the intensity classified DAPI signals. All cell types show a similar profile with a distinct overrepresentation of both markers in low chromatin density classes and a corresponding underrepresentation in high density classes. Note the stronger enrichment of RNA Pol II Ser5P compared to H3K4me3 in class 1 (IC compartment). n = number of analysed nuclei; error bars = standard deviation.
10.1186/s13072-015-0038-0 Comparative topology of SC35 and RNA Pol II Ser5P, markers for transcriptional activity in relation to chromatin density maps. **(A)** 3D-SIM light optical mid-sections from whole 3D acquisitions of nuclei and representative inset magnifications delineating DAPI stained DNA (gray), immuno-stained SC35 (green) and RNA Pol II Ser5P (red). SC35, an integral part of splicing speckles is seen almost exclusively in the IC compartment while RNA Pol II Ser5P shows a preferential localization at decondensed chromatin sites or at the surface of compacted chromatin domain clusters (compare additional file 4). Scale bars: 2 µm; insets 0.5 µm **(B)** *graphs highlighted with yellow background:* relative signal distribution of SC35 (green) and RNA Pol II Ser5P (red) within respective DAPI defined DNA intensity classes. p < 0.001 for DAPI vs. SC35 and RNA Pol II Ser5P, and for SC35 vs. RNA Pol II Ser 5P, except for SC35 vs. RNA Pol II Ser5P in granulocytes (p = 0.004). *Graphs highlighted with light*-*blue background:* quantified levels of relative enrichment (positive values) or depletion (negative values) of SC35 (green) and RNA Pol II Ser5P (red) signals relative to the DAPI signals confirm massive enrichment of SC35 signals in class 1 reflecting the IC compartment. n = number of analysed nuclei; error bars = standard deviation.
10.1186/s13072-015-0038-0 Overview of all measured parameters for a comparative topology in relation to chromatin density maps. *Graphs highlighted with yellow background:* relative **marker** signal distribution within respective DAPI defined DNA intensity classes. *Graphs highlighted with light*-*blue background:* quantified levels of relative enrichment (positive values) or depletion (negative values) marker signals relative to the DAPI signals. This summary demonstrates the similarity of the distributions in all cell types (progenitor = light blue, monoblast = yellow, myeloblast = red, monocyte = green, granulocyte = dark blue). n = number of analysed nuclei; error bars = standard deviation.
10.1186/s13072-015-0038-0 Nucleolar phenotypes during myelopoiesis. Light optical mid-sections of whole 3D-SIM acquisitions show DAPI stained DNA (gray) and nucleoli (green) delineated by a human-anti-nucleolus antibody in representative cell nuclei. Scale bars: 2 µm, insets 0.5 µm. While all cell types contain similar numbers of nucleoli (monoblasts 1-3, myeloblasts 2-3, monocytes 2-4 and granulocytes 1-2; data not shown) they are distinctly shrinked in size in monocytes and in granulocytes compared to their precursors.
10.1186/s13072-015-0038-0 Methods.

## Abbreviations

2D: 2 dimensional
3D: 3 dimensional
3D-FISH: 3D fluorescence in situ hybridization
3D-SIM: 3D structured illumination microscopy
4D: 4 dimensional
7-AAD: 7-aminoactinomycin D
ANC: active nuclear compartment
BAC: bacterial artificial chromosomes
BSA: bovine serum albumin
CB: cord blood
CDCs: chromatin domain clusters
CFEM: correlative fluorescence—electron-microscopic
CTs: chromosome territories
DAPI: 4′,6-diamidino-2-phenylindole
eADS: enhanced absolute 3D distances to surfaces
EM: electron microscopy
ERCB: Emilia Romagna Cord Blood Bank
FCS: fetal calf serum
FISH: fluorescence in situ hybridization
GFP: green fluorescent protein
H3K4me3: histone 3 tri-methylated at lysine 4
H3K9me3: histone 3 tri-methylated at lysine 9
IC: interchromatin compartment
IF: immunofluorescence
IMDM: Iscove’s modified Dulbecco’s medium
INC: inactive nuclear compartment
MFM: multifocus microscopy
mRNPs: messenger ribonucleoprotein particles
NPC: nuclear pore complex
OTFs: optical transfer functions
PBS: phosphate buffered saline
PR: perichromatin region
RI: refractive index
RNA Pol II Ser2P/Ser5P: RNA polymerase II phosphorylated at serine 2/5
RNA Pol II: RNA polymerase II
RNPs: ribonucleo protein particles
SIM: structured illumination microscopy
TAC: tetrameric antibody complex
TEM: transmission electron microscopy
