# Supplementary figures and images for: Remodeling of nuclear landscapes during human myelopoietic cell differentiation maintains co-aligned active and inactive nuclear compartments
Source: Epigenetics Chromatin. 2015 Nov 17;8:47. doi: 10.1186/s13072-015-0038-0 (PMC4647504; doi:10.1186/s13072-015-0038-0)

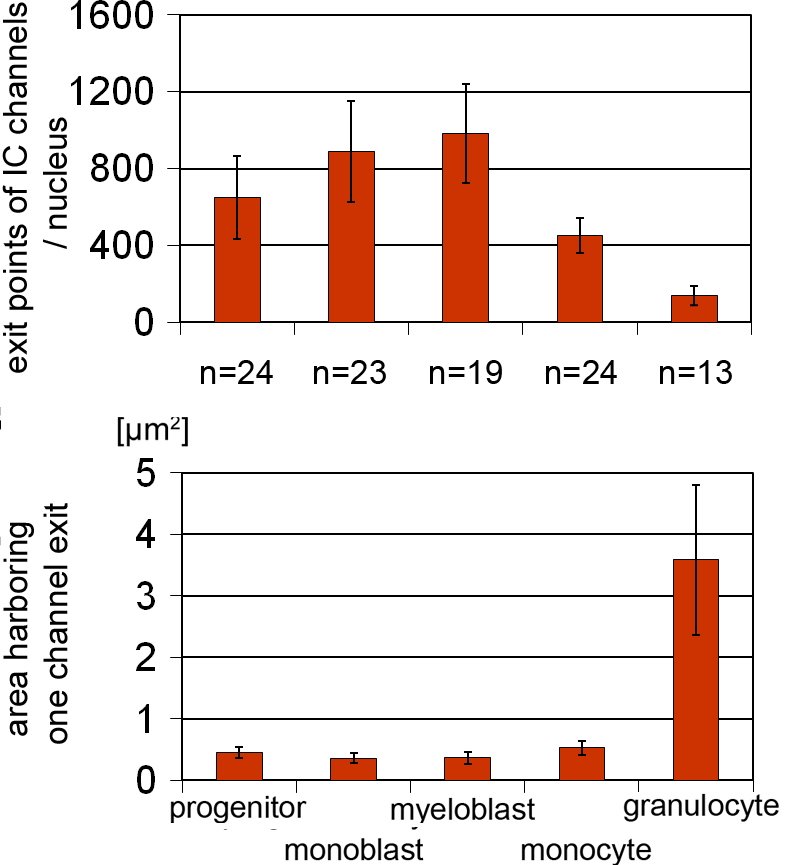

Supplement: Supplementary file 1 — 10.1186/s13072-015-0038-0 Exit points of IC channels at the nuclear surface in myelopoietic cell nuclei. Exit points of IC channels connected to nuclear pores were previously shown to appear as little holes in the nuclear envelope [5,7,23,24]. 3D reconstructions using Amira software (compare Fig. 2B) of whole 3D-SIM 3D acquisitions of DAPI stained nuclei from progenitors, precursors, monocytes and granulocytes were used for a quantitation of such exit points. Upper graph: Number per nucleus; lower graph: average nuclear surface area harboring one exit point. Number of exit points is significantly decreased in monocytes and granulocytes compared to their respective precursors (p < 0.001). Displaying the results as nuclear surface area harboring one channel exit demonstrates the profound difference between granulocytes and the other four cell types (p ≤ 0.001). n = number of analyzed nuclei; error bars = standard deviation. [file 13072_2015_38_MOESM1_ESM.tif]

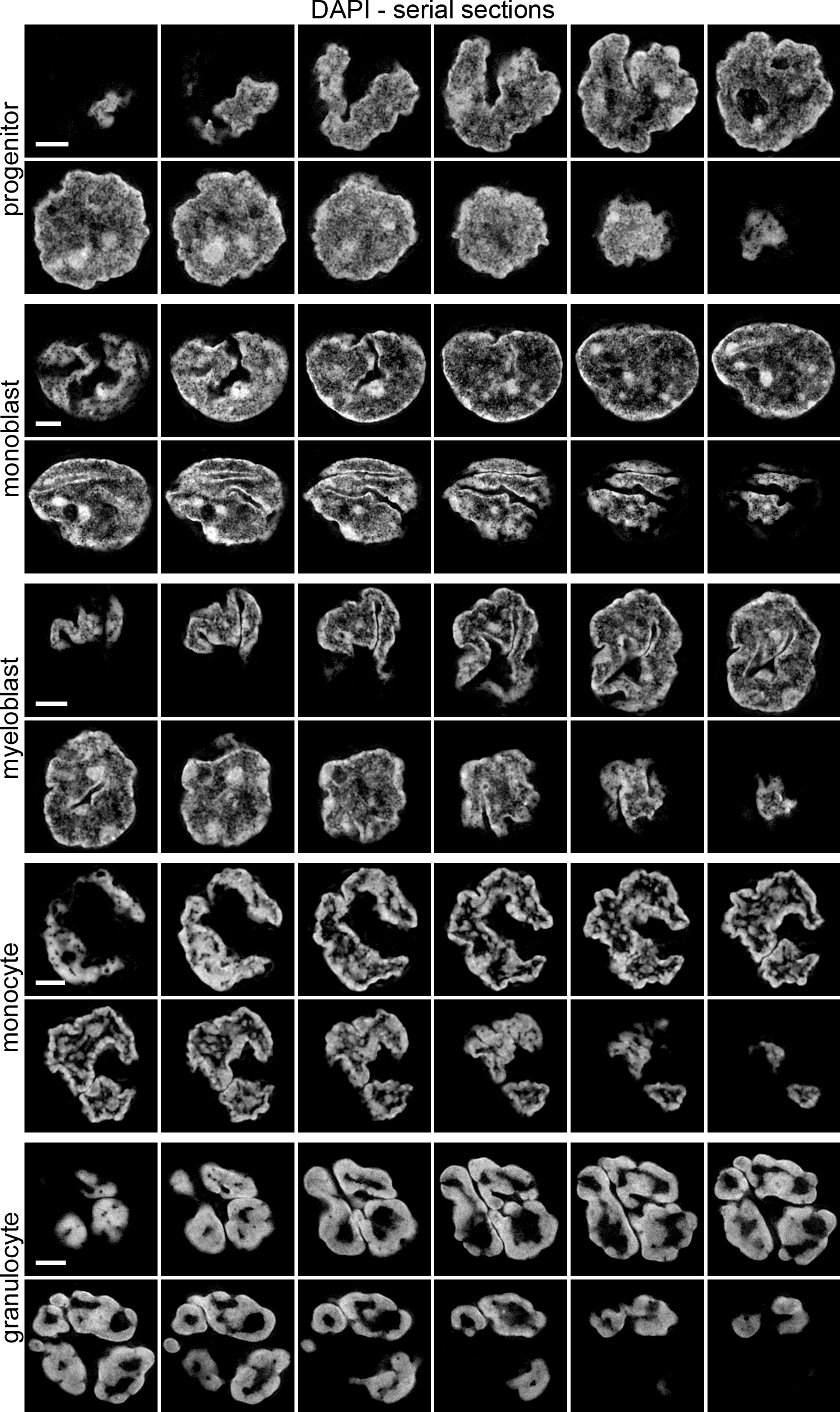

Supplement: Supplementary file 2 — 10.1186/s13072-015-0038-0 Section galleries of nuclei shown in Fig. 2. Galleries of light optical serial sections (axial distance = 125 nm between each optical section) of whole 3D-SIM 3D acquisitions of the DAPI stained nuclei shown in Fig. 2. For the progenitor cell every fifth image (axial distance = 625 nm), for the monoblast every second image (axial distance = 250 nm), for myeloblasts, monocytes and granulocytes every third image (axial distance = 375 nm) is included. Nuclei of progenitors exhibit an overall roundish shape with invaginations at the surface. Monoblast nuclei are of ellipsoid shape with typically deep and complex invaginations. Nuclei of myeloblasts are similar to monoblast nuclei; however, typically they are slightly thicker, and invaginations often pervade the whole nucleus. Monocytes are characterized by horseshoe-shaped nuclei with an irregular surface. Nuclei of granulocytes are divided into several interconnected lobes. [file 13072_2015_38_MOESM2_ESM.tif]

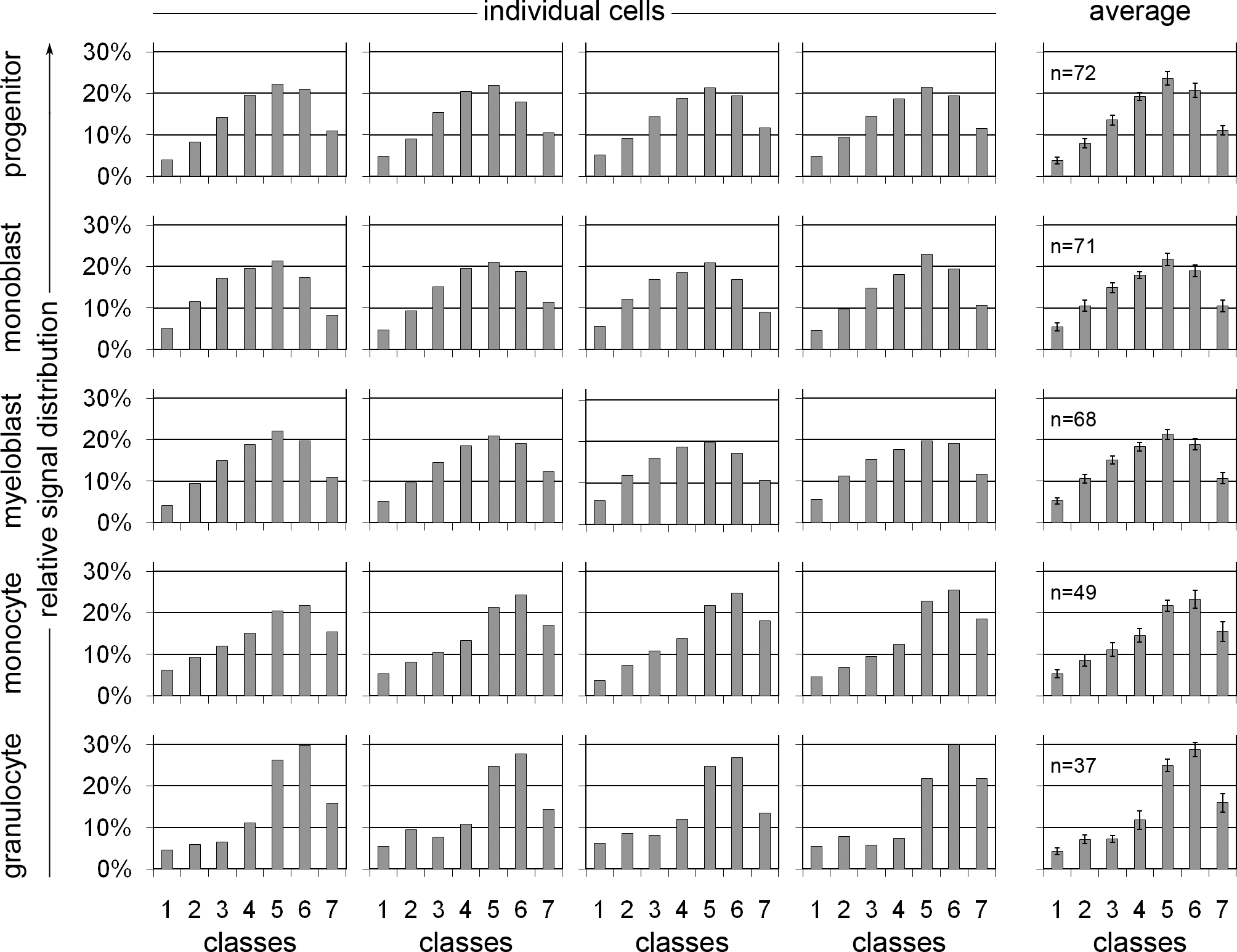

Supplement: Supplementary file 3 — 10.1186/s13072-015-0038-0 DAPI intensity classification profiles from individual nuclei. Four representative chromatin density profiles based on seven DAPI intensity classes are shown for each cell type demonstrating the similarity of profiles within a given cell type and the overall shift towards higher intensity classes in differentiated cell types (monocytes and granulocytes). For comparison the average profiles are repeated from Fig. 4. [file 13072_2015_38_MOESM3_ESM.tif]

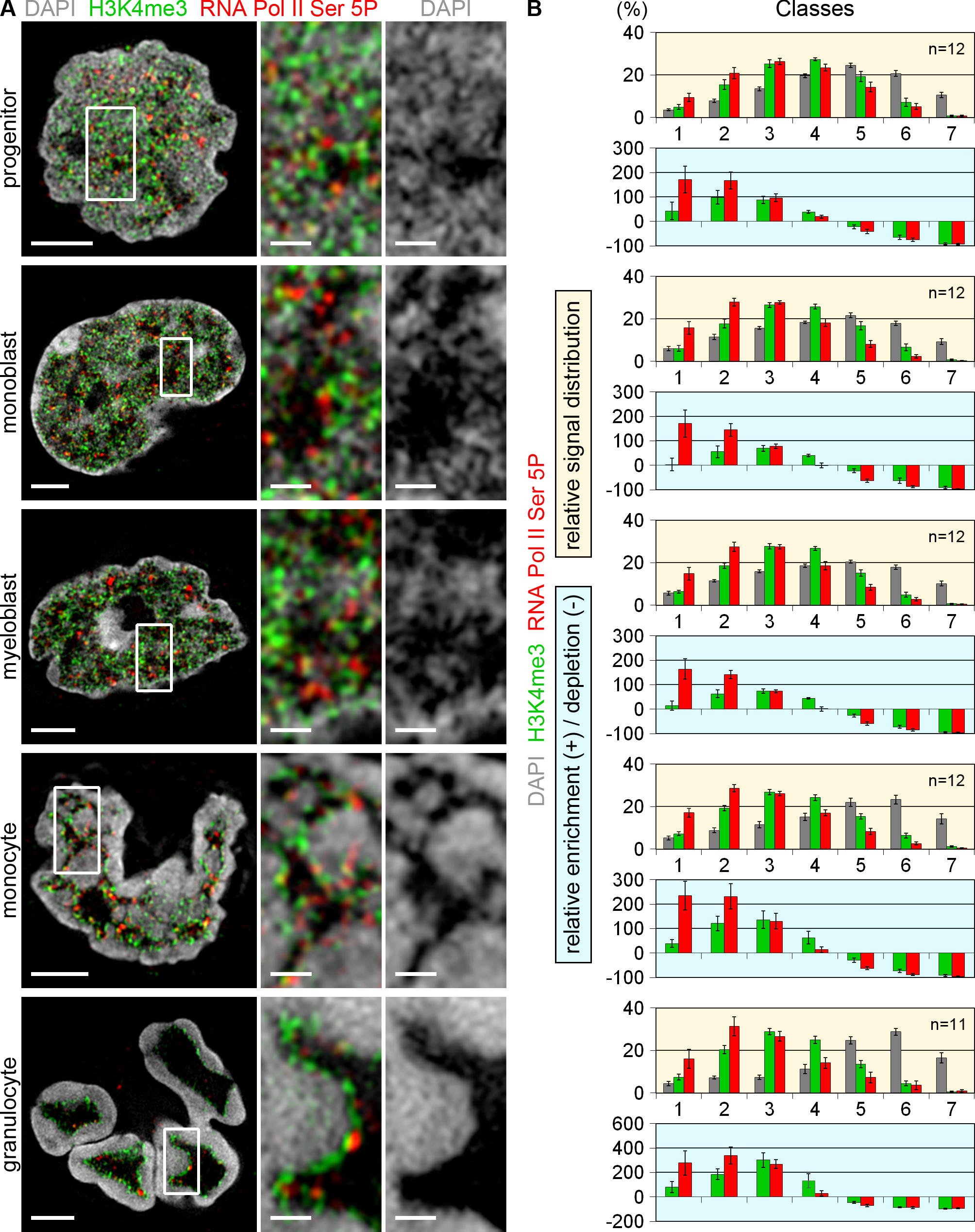

Supplement: Supplementary file 4 — 10.1186/s13072-015-0038-0 Comparative topology of H3K4me3 and RNA Pol II Ser5P, markers for transcriptionally permissive/active chromatin in relation to chromatin density maps. (A) 3D-SIM light optical mid-sections from whole 3D acquisitions of nuclei and representative inset magnifications delineating DAPI stained DNA (gray), immuno-stained H3K4me3 (green) and RNA Pol II Ser5P (red). All cell types show a preferential localization of H3K4me3 and RNA Pol II Ser5P at decondensed chromatin sites or at the surface of compacted chromatin domain clusters. Scale bars: 2 µm; insets 0.5 µm. (B) graphs highlighted with yellow background: relative signal distribution of H3K4me3 (green) and RNA Pol II Ser5P (red) within respective DAPI defined DNA intensity classes. p < 0.005, except for H3K4me3 vs. RNA Pol II Ser5P in progenitors (p = 0.059). Graphs highlighted with light-blue background: quantified levels of relative enrichment (positive values) or depletion (negative values) of H3K4me3 (green) and RNA Pol II Ser5P (red) signals relative to the intensity classified DAPI signals. All cell types show a similar profile with a distinct overrepresentation of both markers in low chromatin density classes and a corresponding underrepresentation in high density classes. Note the stronger enrichment of RNA Pol II Ser5P compared to H3K4me3 in class 1 (IC compartment). n = number of analysed nuclei; error bars = standard deviation. [file 13072_2015_38_MOESM4_ESM.tif]

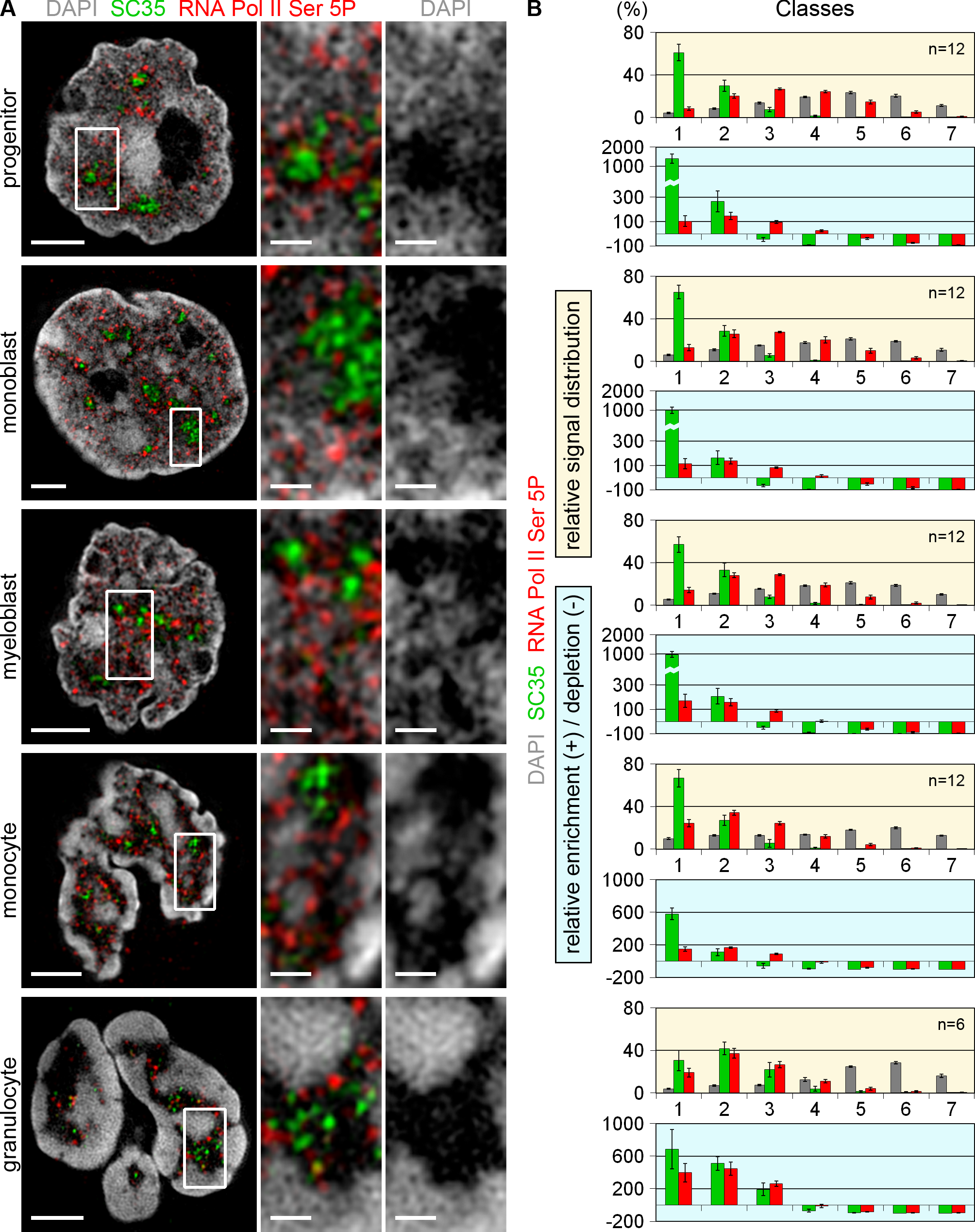

Supplement: Supplementary file 5 — 10.1186/s13072-015-0038-0 Comparative topology of SC35 and RNA Pol II Ser5P, markers for transcriptional activity in relation to chromatin density maps. (A) 3D-SIM light optical mid-sections from whole 3D acquisitions of nuclei and representative inset magnifications delineating DAPI stained DNA (gray), immuno-stained SC35 (green) and RNA Pol II Ser5P (red). SC35, an integral part of splicing speckles is seen almost exclusively in the IC compartment while RNA Pol II Ser5P shows a preferential localization at decondensed chromatin sites or at the surface of compacted chromatin domain clusters (compare additional file 4). Scale bars: 2 µm; insets 0.5 µm (B) graphs highlighted with yellow background: relative signal distribution of SC35 (green) and RNA Pol II Ser5P (red) within respective DAPI defined DNA intensity classes. p < 0.001 for DAPI vs. SC35 and RNA Pol II Ser5P, and for SC35 vs. RNA Pol II Ser 5P, except for SC35 vs. RNA Pol II Ser5P in granulocytes (p = 0.004). Graphs highlighted with light-blue background: quantified levels of relative enrichment (positive values) or depletion (negative values) of SC35 (green) and RNA Pol II Ser5P (red) signals relative to the DAPI signals confirm massive enrichment of SC35 signals in class 1 reflecting the IC compartment. n = number of analysed nuclei; error bars = standard deviation. [file 13072_2015_38_MOESM5_ESM.tif]

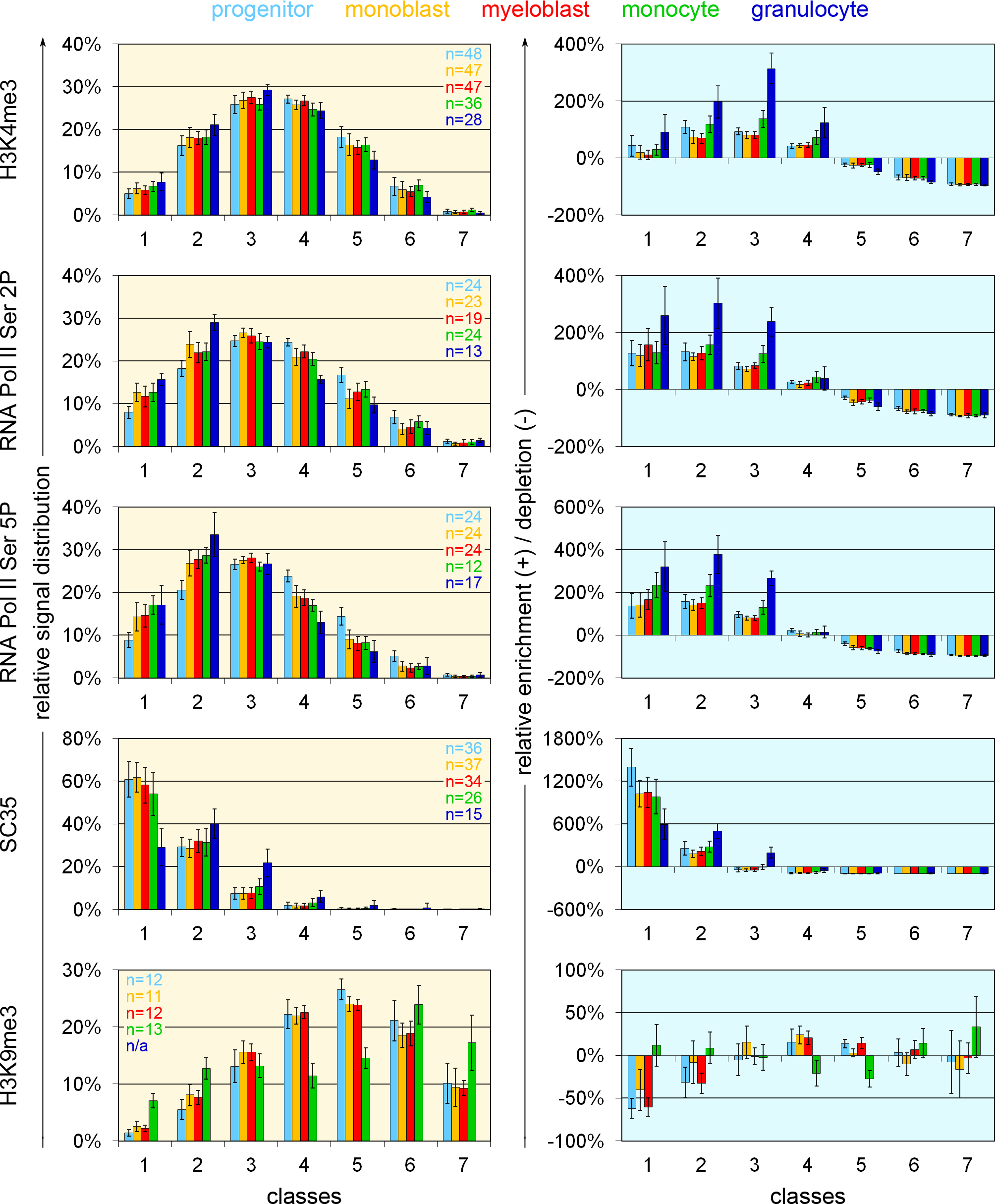

Supplement: Supplementary file 6 — 10.1186/s13072-015-0038-0 Overview of all measured parameters for a comparative topology in relation to chromatin density maps. Graphs highlighted with yellow background: relative marker signal distribution within respective DAPI defined DNA intensity classes. Graphs highlighted with light-blue background: quantified levels of relative enrichment (positive values) or depletion (negative values) marker signals relative to the DAPI signals. This summary demonstrates the similarity of the distributions in all cell types (progenitor = light blue, monoblast = yellow, myeloblast = red, monocyte = green, granulocyte = dark blue). n = number of analysed nuclei; error bars = standard deviation. [file 13072_2015_38_MOESM6_ESM.tif]

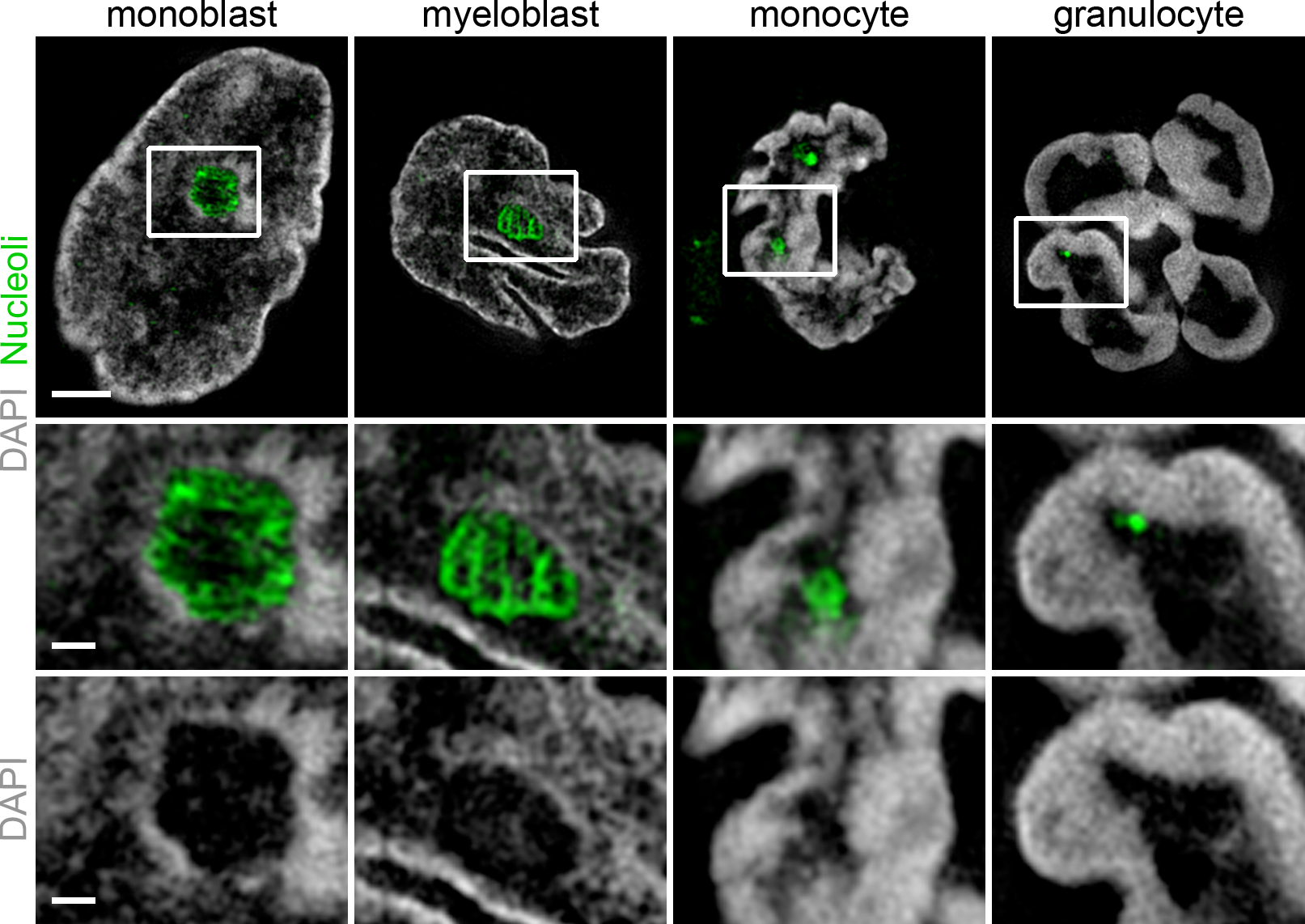

Supplement: Supplementary file 7 — 10.1186/s13072-015-0038-0 Nucleolar phenotypes during myelopoiesis. Light optical mid-sections of whole 3D-SIM acquisitions show DAPI stained DNA (gray) and nucleoli (green) delineated by a human-anti-nucleolus antibody in representative cell nuclei. Scale bars: 2 µm, insets 0.5 µm. While all cell types contain similar numbers of nucleoli (monoblasts 1-3, myeloblasts 2-3, monocytes 2-4 and granulocytes 1-2; data not shown) they are distinctly shrinked in size in monocytes and in granulocytes compared to their precursors. [file 13072_2015_38_MOESM7_ESM.tif]
