# Supplementary material for: Remodeling of nuclear landscapes during human myelopoietic cell differentiation maintains co-aligned active and inactive nuclear compartments
Source: Epigenetics Chromatin. 2015 Nov 17;8:47. doi: 10.1186/s13072-015-0038-0 (PMC4647504; doi:10.1186/s13072-015-0038-0)
Supplement: Supplementary file 8 — 10.1186/s13072-015-0038-0 Methods. [file 13072_2015_38_MOESM8_ESM.docx]

Additional file 8: Methods

Isolation of myelopoietic cells of defined differentiation stages

Human CD34+ cells (progenitors) were purified from umbilical cord blood (CB) samples. In a first step mononuclear cells were isolated by Ficoll-Hypaque (Lymphoprep; Nycomed Pharma) gradient separation and washed 2x with phosphate-buffered saline (PBS), then CD34+ cells were separated using a magnetic cell sorting procedure (EasySep Human CD34 Positive Selection Kit; StemCell Technologies). Briefly, cells were first incubated with a Positive Selection Cocktail, containing a bispecific Tetrameric Antibody Complex (TAC). This complex carries an antibody detecting one specific surface antigen (here CD34) on the one side and an antibody detecting dextran on the other side. Then, magnetic iron beads coated with dextran are added. In addition to binding to the antigen-expressing cells, the TAC now also binds to the magnetic beads. Cells positive and negative for the respective antigen are then separated using a magnet. CD34+ cell purity assessed by flow cytometry was ~95%. The cells were seeded in 6-well plates at 1x10^6^ cells/ml in Iscove’s modified Dulbecco’s medium (IMDM) (GIBCO) containing 20% Human Serum (Bio-Whittaker), SCF (50 ng/ml), Flt3-ligand (Flt3L) (50 ng/ml), TPO (20ng/ml), IL-6 (10 ng/ml) and IL-3 (10 ng/ml) (all from R&D Systems,) and were allowed to recover over night before being processed for IF or TEM.

Myeloid precursors (myeloblasts and monoblasts) were obtained by in vitro differentiation of CB derived CD34+ cells. CB CD34+ cells were cultured in IMDM supplemented with 20% FCS (Bio-Whittaker) and human hematopoietic cytokines: SCF (50 ng/ml), Flt3-ligand (Flt3-l) (50 ng/ml), IL-6 (10 ng/ml) and IL-3 (10 ng/ml) (all from R&D Systems). After 7 days of culture, CD14+ (monoblasts) and CD14- (myeloblasts) cell fractions were separated by a magnetic cell sorting procedure (EasySep Human CD14 Positive Selection Kit; StemCell Technologies). Analysis by flow cytometry for CD14 antigen expression estimated at about 15-20% of the entire cell population. After separation cells were seeded in 6-well plates at 1x10^6^ cells/ml and allowed to recover O/N before being processed for IF or TEM.

Monocytes were isolated from peripheral blood using either a magnetic cell sorting procedure (EasySep Human CD14 Positive Selection Kit; StemCell Technologies) or Histopaque gradients (Sigma-Aldrich) according to the manufacturer’s guidelines. Typical monocytes with horseshoe-shaped nuclei were about 5-10% of the entire cell population. Monocytes isolated using immuno-magnetic separation were allowed to recover over night, monocytes isolated using Histopaque gradients were processed immediately for IF or TEM.

Granulocytes were isolated from peripheral blood using Histopaque gradients (Sigma-Aldrich) according to manufacturer’s guidelines and processed immediately for IF, FISH or TEM. Granulocyte cell purity was >98%.

*Immunodetection*

For immunofluorescence (IF) detection, high precision borosilicate glass coverslips, no. 1.5 (170 µm ± 5 µm thickness, Carl Roth) were coated for 45-60 min with 1.25 mg/ml poly-lysine, washed 3x5 min with H_2_O_dest_ and air dried. 150.000 – 300.000 cells in 150-300 µl medium were pipetted onto a coverslip and cells were allowed to attach for 30-60 min (granulocytes), 1 h (monocytes), 2 h (monoblasts, myeloblasts) or 3 h (progenitors) prior to fixation. Cells were washed with PBS and fixed with 2% paraformaldehyde/PBS for 8 min followed by 2x 5 min washing with PBS. The samples were quenched with 20 mM glycine/PBS for 10 min, washed with PBS, permeabilized with 0.3% Triton X-100/PBS for 10 min and subsequently incubated in blocking buffer (150 mM NaCl, 15 mM Hepes/KOH, 2 mM MgCl_2_, 0.1 mM EGTA, 0.2% Triton X-100, 0.5% fish skin gelatin, 2% BSA) for 1.5-2 h. Antibodies were diluted in blocking buffer and incubated for 1.5-2 h (primary) or 45-60 min (secondary) followed by washing 6x with blocking buffer. Cells were washed 2x with PBS, postfixed with 4% paraformaldehyde/PBS for 10 min and washed 2x with PBS. DNA was counterstained with 2.5 µg/ml 4',6-diamidino-2-phenylindole (DAPI) in PBS for 8 min. Samples were washed 5x with PBS, mounted in Vectashield antifade mounting medium (Vector Laboratories) and sealed with nail varnish.

*Sample preparation for TEM and osmium ammine B staining*

Cells were seeded onto round 12 mm coverslips (ScienceServices) as described above for immunodetection. For fixation under normotonic conditions cells were washed with PBS and fixed with 4% paraformaldehyde/PBS for 10 min, for fixation under hypotonic conditions cells were incubated in 0.3x PBS for 1 min and fixed with 4% paraformaldehyde/0.3x PBS for 10 min. In both cases fixation was followed by 2x 5 min washing with PBS. Fixed samples were dehydrated in an ascending ethanol series: 2x 5 min 30% on ice, 2x 5 min 50% on ice, 2x 10 min 70% on ice, 2x 10 min 90%, 3x 10 min 100%. The coverslips were transferred into embryo glass dishes, the ethanol was exchanged to LR white resin (medium grade, resin only; ScienceServices) and the samples were moved around until no clouds of ethanol were observed anymore. After a minimum of 30 min incubation the resin was replaced with fresh resin, the samples were moved around again and incubated at 4°C over night. After removal of the lid Beem capsules size “00” (EMS) were filled with fresh LR white resin and the coverslip with the cells was place on top, with the cells facing down on the resin. The capsule with the coverslip was inverted, placed on aluminium foil and polymerized at 60°C for 48 h.

The coverslip was detached using liquid nitrogen, the capsule was removed and the block was trimmed to a surface area of about 1x1 mm using razor blades. Ultrathin section (100 nm) were cut with a diamond knife (Diatome) on a Leica Ultracut UCT ultramicrotome. Sections were picked up on 300 mesh gold grids (Plano) cleaned by washing briefly with 0.25 N HCl, drying on filter paper, washing briefly with acetone, drying again, or on 2x1 mm slot gold grids coated with Formvar/carbon (ScienceServices). Both grid types were floated on H_2_O_bidest_ for about 40 min prior to use to reduce their charge. After complete drying of the grids (overnight) DNA was stained with osmium ammine B (Polysciences) largely according to [65]. Grids with sections facing down were floated on freshly prepared 5 N HCl for 40 min, followed by 30 min incubation on freshly prepared osmium ammine B staining solution (0.2% (=2.8 mM) osmium ammine B, 0.2 N HCl, 200 mM Na_2_S_2_O_5_) and washing on H_2_O_bidest_ for 5-10 min. Subsequently the samples were dried over night.

*3D-FISH and 3D distance measurement of fluorescent signals to the nuclear border*

Preparation of complex DNA probes and 3D-FISH: Human BAC clones for chromosomes 1 and 12, were verified by testing their correct position on metaphase chromosomes and by the comparison of restriction patterns, mostly SWAI and EcoRV (New England Biolabs), with in silico patterns obtained by the software pDRAW32 (http://www.acaclone.com). Genomic DNA from pooled BAC probes was amplified by isothermal whole genome amplification using the “genomi_Phi” kit from GE Healthcare and labeled with haptens by nick translation. Approximately 40 ng of labeled probe/µl hybmix was used. Hybridization setup and detection of probes was performed as described in detail in [66].

*3D distance measurement of fluorescence signals to the border of nuclear lobes in granulocytes:*

An in house developed software was used for the automatic measurement of the shortest absolute 3D distances of all BAC signals to the surface of the nuclear border (enhanced absolute 3D distances to surfaces, **eADS,** previously described in [38]). The surface of the reference structure (here: DAPI stained nuclear lobes of granulocytes) was determined by applying a user set threshold: all voxels with intensities below the set threshold were set to zero. The surface of the reference structure contains all voxels with an intensity >0, which are directly adjacent to at least one voxel with an intensity = 0. The program measures the shortest 3D distance of all voxels of a BAC signal to the surface of the reference structure by calculating the euclidean distances between the voxels of a BAC signal and the voxels of the surface of the reference structure (considering the voxel size in the spatial directions). All signals that are located inside the volume surrounded by the surface are assigned as negative values, while signals that are located outside of the surface have positive distance values. Measured distances were divided into user defined classes (at least z-voxel size), the relative frequency in every class is determined by the summed up intensity value of the signal voxels which are allocated to this class. The mean values of the classes for all evaluated nuclei were plotted as a graph.
